# Evolution of the Automatic Rhodopsin Modeling (ARM) Protocol

**DOI:** 10.1007/s41061-022-00374-w

**Published:** 2022-03-15

**Authors:** Laura Pedraza-González, Leonardo Barneschi, Daniele Padula, Luca De Vico, Massimo Olivucci

**Affiliations:** 1 Dipartimento di Biotecnologie, Chimica e Farmacia, Università degli Studi di Siena, Via Aldo Moro 2, 53100 Siena, Italy; 2 Department of Chemistry, Bowling Green State University, Bowling Green, OH 43403 USA; 3 Present Address: Department of Chemistry and Industrial Chemistry, University of Pisa, Via Moruzzi 13, 56124 Pisa, Italy

**Keywords:** QM/MM, Rhodopsins, Photochemistry, Photobiology, Python package

## Abstract

In recent years, photoactive proteins such as rhodopsins have become a common target for cutting-edge research in the field of optogenetics. Alongside wet-lab research, computational methods are also developing rapidly to provide the necessary tools to analyze and rationalize experimental results and, most of all, drive the design of novel systems. The Automatic Rhodopsin Modeling (**ARM**) protocol is focused on providing exactly the necessary computational tools to study rhodopsins, those being either natural or resulting from mutations. The code has evolved along the years to finally provide results that are **reproducible** by any user, **accurate** and **reliable** so as to replicate experimental trends. Furthermore, the code is **efficient** in terms of necessary computing resources and time, and **scalable** in terms of both number of concurrent calculations as well as features. In this review, we will show how the code underlying ARM achieved each of these properties.

## Introduction: Contents and Scope

**Fig. 1:**
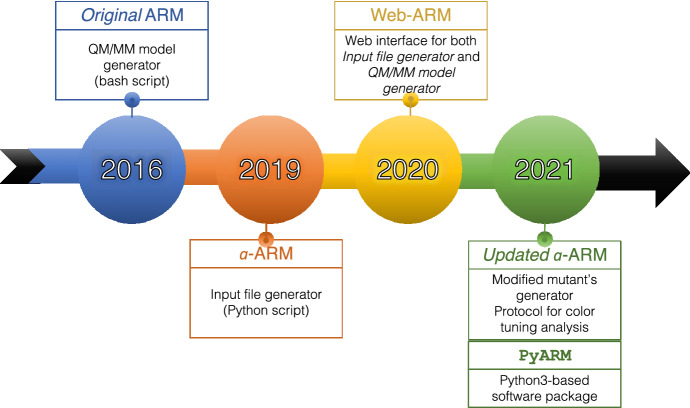
Timeline for Automatic Rhodopsin Modeling (ARM) development detailing various parts of the ARM protocol and the years of the corresponding publications

This review deals with the development of the Automatic Rhodopsin Modeling protocol (ARM), as seen in the last 5 years (Fig. 1). ARM represents a protocol capable of reliably building and analyzing computational models of rhodopsins, a superfamily of photoactive proteins. Given their major role in many aspects of life, from the basic act of ion-gating and ion-pumping in Archaea and Eubacteria to vision in animals, rhodopsins are currently studied extensively. Furthermore, rhodopsins and their engineered mutants represent powerful tools for potential biotechnological applications, the most prominent of which is optogenetics. Thus, researchers are constantly looking for new rhodopsins with particular photochemical properties, those being, among others, a specific absorption wavelength, a long or short excited state lifetime, and a strong fluorescence. Section 2 gives a panoramic view of rhodopsins and discusses current and future technological applications.

ARM was developed to provide a basic quantum-mechanics molecular mechanics (QM/MM) computational model, but sufficiently accurate so that differences in the model would reflect actual changes in the behavior of a different/mutated rhodopsin, and vice versa. This first iteration of ARM, called ARM_original_, was created to generate models capable of accurately reproducing the spectral trends observed for a limited set of rhodopsins, and will be described in Sect. 3. In particular, ARM_original_ models have been shown to be capable of reproducing trends in light absorbance maximum values in rhodopsin of different origins, and provide effective tools to discern the causes of effects such as blue- or red-shifting of the absorbed wavelength.

The natural extension of the ARM code has been to extend the general accuracy and applicability of the models and, most importantly, the level of automation in building protocol. Section 4 presents the *advanced* Automatic Rhodopsin Model building protocol (*a*-ARM), which meant completely rewriting the previous ARM code, and incorporating the possibility to automatically prepare inputs for the protocol itself. The completely new input preparation phase removed the need for user files manipulation and possible source of errors, hence achieving **reliability** and complete **reproducibility** of the results. The code behind *a*-ARM has also been used to power a web-interface, which allows, in principle, any user to obtain rhodopsin models of the same quality (Web-ARM, Sect. 5). This meant the extension of the benchmarking pool to more rhodopsins, thus increasing the applicability of the protocol, which, in turn, made it possible to use ARM models to guide the rational design of rhodopsins. More specifically, *a*-ARM can now be used to suggest which residue to mutate to obtain, for instance, a desired spectroscopic effect.

Finally, the ARM code was encapsulated inside a python-based package, called PyARM and described in Sect. 6. While *a*-ARM was already efficient in producing a single rhodopsin model, PyARM allows hundreds of concurrent rhodopsin models to be obtained automatically. This high level of efficiency is obtained by allowing the code to completely take care of all necessary calculations (complete automation), through the clever use of a highly modular code structure. Different types of analyses are now possible, all through the use of automatic, user-friendly, command-line Python drivers. Finally, the modular nature of PyARM allows the easy implementation of additional features, thus scaling-up the usability of the code with additional features.

## Rhodopsins: a Family of Biological Photoreceptors

### Structure and Diversity

**Fig. 2:**
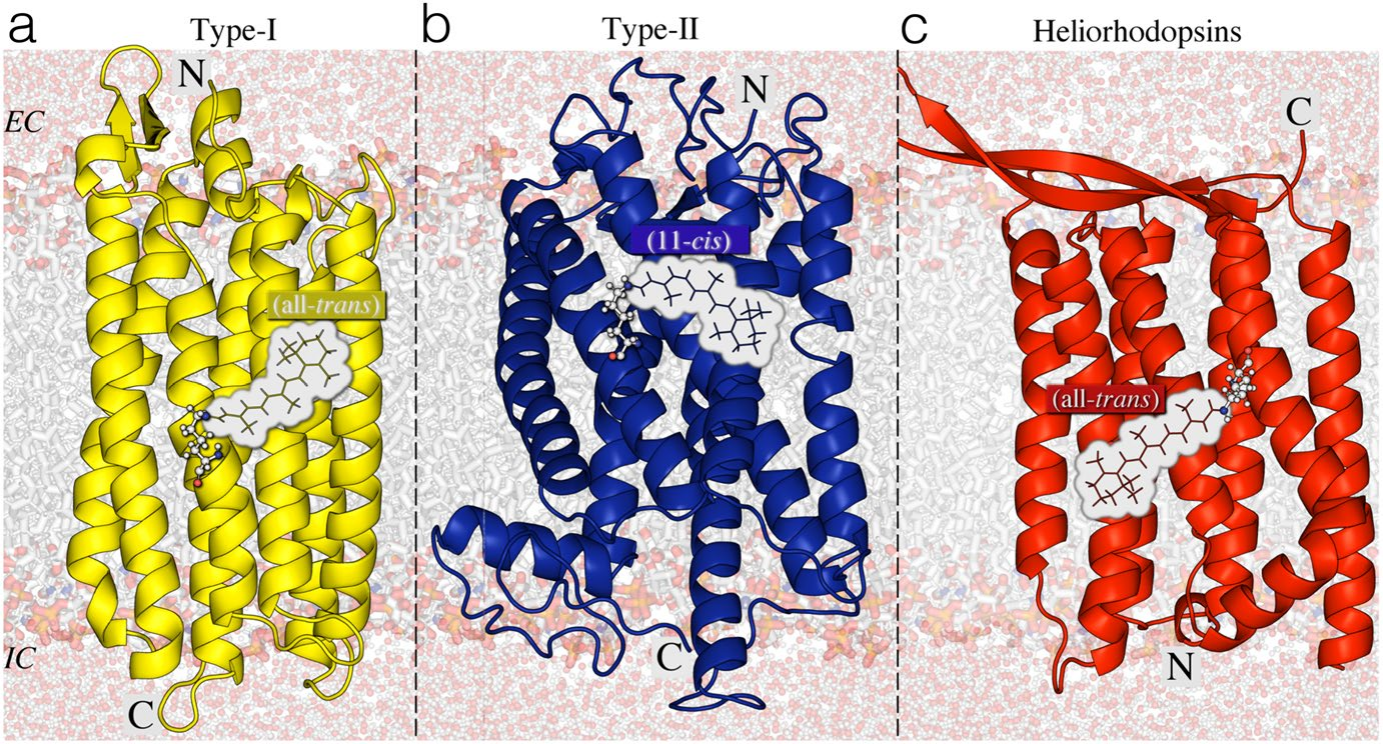
**a**–**c** Rhodopsin types: structural similarities and differences. Rhodopsin proteins are classified into three types: yellow cartoon microbial or type-I, blue cartoon animal or type-II, red cartoon heliorhodopsins. For each type, the retinal chromophore in the $\text{dark adapted state (DA)}$ is presented as lines and the covalently linked lysine is shown as ball-and-sticks. The orientation of the N terminus and C terminus residues with respect to the extracellular (EC) and intracellular (IS) surfaces of the membrane is specified. The structures correspond to **a** KR2 (type-I) [6REW [1]], **b** Rh (Type-II) [1U19 [2]] and **c** TaHeR (heliorhodopsin) [6IS6 [3]]

**Fig. 3:**
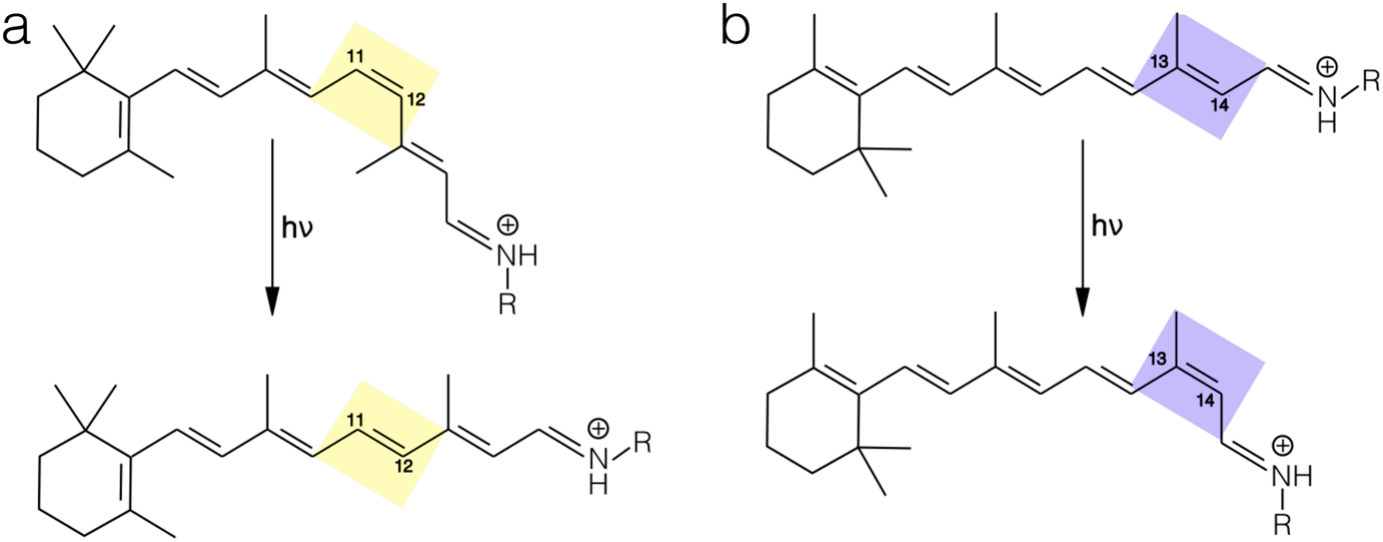
Primary photoreaction in animal, microbial and helio-rhodopsins. Retinal isomerization from **a** the $11-{cis}$ to the $\text{all-}{trans}$ form and **b** from the $\text{all-}{trans}$ to the $13-{cis}$ form is the primary reaction in animal and microbial/helio-rhodopsins, respectively

Rhodopsins constitute a vast family of photoreceptive, membrane-embedded, seven-helix proteins, structurally formed by an opsin apoprotein and a retinal that serves as a chromophore^1^ to absorb photons for either energy conversion or the initiation of intra- or intercellular signaling. As illustrated in Fig.  2, the opsin features an internal pocket hosting the retinal chromophore, which is covalently attached by a Schiff base linkage to the $\varepsilon$-amino group of a lysine side-chain in the middle of helix VII; the resulting retinal Schiff base $({{r}\text{SB}})$ is protonated in most cases, constituting the so-called retinal protonated Schiff base $({r}\text{PSB})$, illustrated in Fig. 3. Changes in protonation state of the ${r}\text{SB}$ are crucial for both signaling and transport activity of rhodopsins [5–7].

Recent genomic advances have revealed that tens of thousands of rhodopsin genes are distributed widely in all domains of life (i.e., *Eukaryotes*, *Bacteria*, and *Archaea*) [5–10], and reside in many diverse organisms such as animals (e.g., vertebrates and invertebrates), microorganisms, and even viruses [11, 12]. Based on their host organism, rhodopsins are divided into different types (see Fig. 2): animal (type II) rhodopsins, a specialized subset of $\text{G-protein-coupled receptor (GPCR)}$; microbial (type I) rhodopsins [6]; and heliorhodopsins—a recently discovered new type of light-sensing microbial rhodopsins [13].

Although members of the three types display a remarkably constant general architecture, they exhibit large differences in amino acid sequence (i.e., they have almost no sequence homology), as reflected in different chromophore cavities as well as in certain structural features [5, 7, 8, 13, 14]. In animal rhodopsins, the equilibrium $\text{dark adapted state (DA)}$ shows a 11-*cis* (i.e., C11–C12 double bond) $r\text{PSB}$ chromophore, which photoisomerizes to its $\text{all-}{trans}$ configuration (Fig. 3a). In both microbial and heliorhodopsin families the $\text{DA}$ state is dominated by an $\text{all}-{trans}$ (i.e., C13–C14 double bond) $r\text{PSB}$ chromophore, which is usually transformed into the $13-{cis}$ configuration (see Fig.  3b) upon light absorption. Finally, in heliorhodopsins, the N-terminus amino acid is exposed to the cytoplasm or intracellular (IC) part, and the C-terminus residue faces the extracellular (EC) part of the cell membrane, whereas the opposite happens for the other two families (Fig. 2) [15].

### Biological Functions

Rhodopsins exhibit an extensive pool of biological functions [5, 6, 8, 9, 16, 17]. For instance, animals use the photosensory functions (i.e., visual responses) of type II rhodopsins, lower organisms utilize type I for light energy conversion and intracellular signaling, while the function of heliorhodopsins is still not well elucidated [18–20].

In particular, microbial rhodopsins (type I) are represented in many diverse microorganisms, spanning the three domains of cellular life, as well as in giant viruses [12]. As such, modifications of a single protein scaffold through evolution produced many novel, chemical, light-dependent biological functions [5–7, 9, 10, 15, 17, 21]. Such functions can be roughly divided into two categories: (1) photoenergy transducers able to convert light into electrochemical potential to energize cells (e.g., light-driven ion pumps catalyzing outward active transport of protons [H$^+$], inward chloride transport [Cl$^-$], and outward sodium transport [Na$^+$]); and (2) photosensory receptors that make use of light to gain information about the environment to regulate inter- or intracellular processes (e.g., photosensors with membrane-embedded or soluble transducers, ion channels [anion and cation channel rhodopsins (ChRs)], and enzyme rhodopsins) [5–7, 9, 17, 22].

### Photoreactivity

Rhodopsin functions are triggered by highly stereo-selective photoisomerization events, i.e., generally *cis* $\rightarrow$ *trans* of the C11=C12 bond for type II and *trans* $\rightarrow$ *cis* of the C13=C14 for type I and heliorhodopsins (Fig.  3). The specific wavelength (hereinafter referred to as $\text{Maximum absorption wavelength}$ ($\lambda _{\max }^{a}$)) is due to a phenomenon called “opsin-shift”, which consists of spectral shifts, in a wide range of the UV/visible spectra, modulated by the interaction between the retinal chromophore and the opsin residues.

**Fig. 4:**
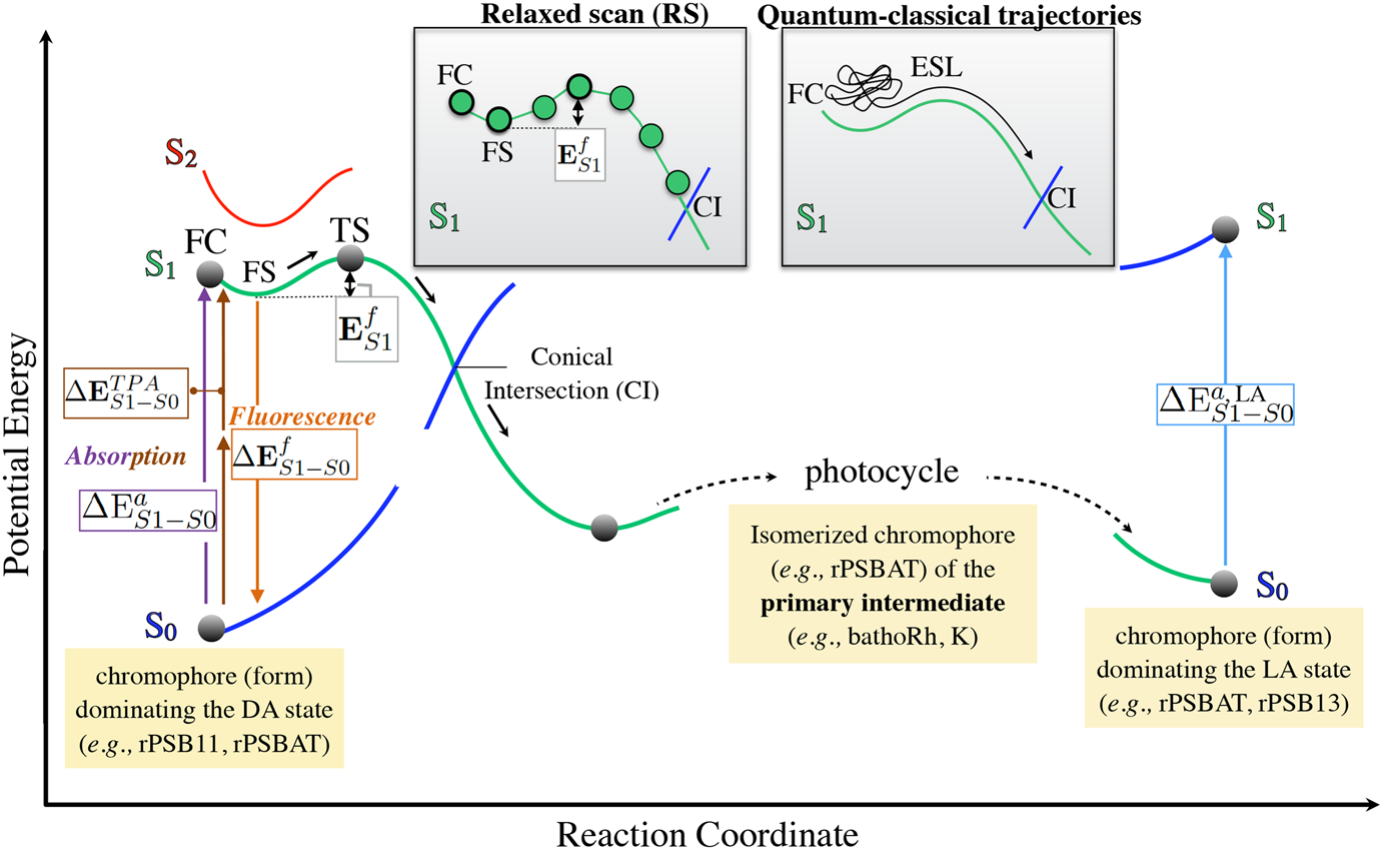
Light-induced and light-emission properties of rhodopsin proteins, investigated using computational modeling. Schematic diagram displaying the photoiomerization path, including the relevant $\text {S}_{0}$, $\text {S}_{1}$ and $\text {S}_{2}$ energy profiles of a generic rhodopsin. $\text{QM/MM}$ models are used to compute the vertical excitation energy for absorption ($\Delta \text {E}_{S1-S0}^{a}$ $\equiv$ $\lambda _{\max }^{a}$), fluorescence ($\Delta \text {E}_{S1-S0}^{f}$ $\equiv$ $\lambda _{\max }^{f}$) and two-photon absorption ($\Delta \text {E}_{S1-S0}^{\text {TPA}}$ $\equiv$ $\lambda ^{\text {TPA}}_\text {max}$) as well as the excited state $\text{energy isomerization barrier}$ $(\text {E}^{f}_{S1})$ associated with emission, computed as the energy difference between the $\text{fluorescent excited-state (FS)}$ structure and the transition state (TS).* Inset *(top-center) Schematic illustration of the calculation of an excited state isomerization path providing the $\text {E}^{f}_{S1}$ value via a $\text{Relaxed scan (RS)}$ and of a Franck–Condon (FC) quantum-classical trajectory (this provides, in case of an ultrafast reaction, an estimate of the $\text{ESL}$ associated to the double bond $\text{energy isomerization barrier}$). $\text{QM/MM}$ models can also been used to investigate the structure and spectroscopy of primary photocycle intermediates (batho and K intermediates) and of photocycle intermediates corresponding to light-adapted states

Figure 4 depicts the inter-state photoisomerization pathways of rhodopsins, representing the potential energy change, i.e., the progression along the potential energy surface $\text{(PES)}$, along a reaction coordinate possibly involving a combination of electronic transitions between the $\text{Ground-state}$ $(\text {S}_{0})$ and both first ($\text {S}_{1}$) and second ($\text {S}_{2}$) excited electronic states. Such a coordinate is usually considered as the skeletal dihedral angle of the double-bond highlighted in Fig.  3. The involved electronic states commonly change their electronic character and can cross [23, 24]. Different rhodopsin “functions” may take advantage of different features of the rhodopsin $\text{PES}$, illustrated in the following.

The primary event of light absorption of one photon of energy $h\nu$ prompts the vertical electronic $\pi$-$\pi ^{*}$ transition of the retinal chromophore from the ground to the first excited Franck–Condon^2^ ($\text{FC}$) state. The length of the $\pi$-conjugated polyene chain in the retinal chromophore, and the protonation state of the ${r}\text{SB}$ linkage, determine the energy gap, hereafter called $\text{Vertical Excitation energy}$ $(\Delta \text {E}_{S1-S0}^{a})$, of this process [5]. The computational evaluation of $\Delta \text {E}_{S1-S0}^{a}$ allows the prediction of $\lambda _{\max }^{a}$ for a given rhodopsin, and, in general, for the modeling of light absorption. Considering that the wavelength dependence of the absorption efficiency defines the colors of the rhodopsin proteins, the modulation of the $\Delta \text {E}_{S1-S0}^{a}$ gap makes possible the engineering of either blue- or red-shifted rhodopsin variants, absorbing in specific regions of the UV-Vis spectrum (i.e., color tuning, see Sect. 6.4) [19–21, 25–35]. Photoexcitation to the $\text {S}_{1}$ state can also be achieved by simultaneous absorption of two infrared photons (i.e., $\text{Two-Photon Absorption} \,(\text{TPA})$ process) of the same energy $\Delta$E$^{\text {TPA}}_\text {S1-S0}$, corresponding to half of the energy necessary for $\text{One-Photon Absorption (OPA)}$ [23, 36–40].

After photoexcitation, the retinal chromophore leaves the $\text{FC}$ region by relaxing along stretching and torsional modes, and starts the exploration of the $\text {S}_{1}$ $\text{PES}$. Depending on the surface topography, it may (1) quickly encounter a minimum, hereinafter called $\text{fluorescent excited-state (FS)}$, or (2) continue visiting other regions of the $\text {S}_{1}$ $\text{PES}$. In (1), the chromophore remains in a long-lived excited state that eventually decays back to the ground state via fluorescence, i.e., spontaneous emission of radiation (luminescence) [4]. In this case, a photon of a different wavelength can be emitted after a short while (10$^{-9}$ to 10$^{-5}$ s). The energy gap associated to this process is denominated $\text{Vertical Emission energy}$ $(\Delta \text {E}_{S1-S0}^{f})$. Therefore, the computational evaluation of $\Delta \text {E}_{S1-S0}^{f}$ allows the prediction of the $\text{Maximum emission wavelength}$ $(\lambda _{\max }^{f})$ for a given rhodopsin, and in general the modeling of emission properties, such as fluorescence. In (2), that is, if there is a shallow or no $\text{FS}$, the retinal chromophore twists around the reactive C=C bond and reaches the $\text{Conical intersection (CI)}$ region, where it decays to $\text {S}_{0}$. As illustrated in Fig. 4, “reacting” rhodopsins then trigger a series of sequential protein moiety conformational changes (required for their biological functions) [8, 10, 23] and returns to the initial state (e.g., process known as photocycle), whereas the “non-reacting” molecules relax back to the original $\text {S}_{0}$ state without entering such a photocycle. The described cycle allows microbial rhodopsins to repeat their functions every light stimulation since the chromophore is ultimately regenerated through the photocycle. This type of photocycle (in chemistry one would talk about type 1 photochromics) is remarkably different from that of vertebrate visual opsins (i.e., vertebrate rhodopsin and cone visual pigments), in which the retinal chromophore dissociates after the photoreaction and, therefore, additional retinal is required to regenerate the pigments [9].

### Applications of Natural and Engineered Rhodopsins: Optogenetics

The modeling of primary photoproducts, photoisomerization reaction paths and bistable states in animal and microbial rhodopsins is of great interest for engineering photoswitchable fluorescent probes [41–43]. Bistable rhodopsins are rhodopsins featuring two stable isomeric forms (i.e., characterized by two chromophore isomers such $\text{all}-{trans}$ and $13\text{-}{cis}$) and thus require the sequential and independent absorption of two photons (often of different wavelengths) to complete the photocycle [43]. Certain bistable rhodopsins can be interconverted using light of different wavelengths (type-2 or P-type photochromism^3^) [44]. However, more frequently the $\text{light adapted state (LA)}$ state reverts back to the $\text{DA state}$ state thermally (type-1 or T-type photochromism), in which case (i.e., for monostable microbial rhodopsins) the photocycle is completed after the absorption of only one photon [44]. Applications of bistable rhodopsins are related to different properties/functions of the DA and LA states and the use of light irradiation to change the rhodopsin isomeric composition passing from a $\text{DA}$-dominated to a $\text{LA}$-dominated equilibrium. Since the efficiency of such conversion is proportional to the difference between the two $\lambda _{\max }^{a}$ values, it is apparent that achieving bistable rhodopsins featuring one form with a $\lambda _{\max }^{a}$ value significantly shifted to the red, may facilitate applications where it is important to switch on-and-off (i.e., control) the rhodopsin biological function using light irradiation [43].

The photoisomerization event that initiates each rhodopsin function (see Sect. 2.2), has been studied widely in scientific areas such as physics, chemistry, and biology [5–7], with the aim of allowing for novel and modern applications in fields as diverse as medicine [45–47], bioenergetics [9, 48], biotechnology and neurosciences [7–10, 16], among others [9]. Particularly, specific microbial rhodopsins that function as either ion transporters or channels are at the heart of a new biotechnology called optogenetics.

Optogenetics (i.e., a combination of “optics” and “genetics”) uses mainly genetically encoded, and often specifically engineered, microbial rhodopsins for the optical control of physiological processes [7–10, 16, 41]. The development of so-called optogenetic tools leads to the investigation of the nervous system at the cellular and tissue level, without noticeable tissue damage as well as side effects. Currently, rhodopsins with potential application as optogenetic tools are used as light-driven actuators (i.e., action potential triggers), light-driven silencers (i.e., action potential quenchers) and as fluorescent reporters (i.e., action potential probes) [7].

In order to either improve the current or develop new optogenetic tools, it is imperative to gain further insight into the different factors controlling color tuning in rhodopsins, to then be able to increase the variety of $\lambda _{\max }^{a}$, thus enabling simultaneous optical control by different colors of light [19–21, 25–35]. As such, various rhodopsin genes have been screened in order to find additional colors [49, 50]. In particular, while many blue-absorbing rhodopsin at $\lambda _{\max }^{a}$ < 500 nm have been reported [51] and even applied to optogenetics [49], the longer absorption maxima are limited in $\lambda _{\max }^{a}$ < 600 nm. In this regard, there is presently an interest in screening rhodopsin variants exhibiting a longer $\lambda _{\max }^{a}$ and/or enhanced fluorescence (i.e., high fluorescence intensity) [52], achieved through the effects of single or multiple amino acid mutations of a template $\text{Wild-type (WT)}$ structure.

The rational design of artificially mutated variants is necessary to identify the amino acid replacements that are effective for color tuning and for influencing fluorescent properties. A systematic experimental screening of thousands of possible candidates is not feasible, thus requiring the development of computational approaches for a fast, congruous, and rational design of in silico point mutations, to narrow down the number of tested candidates.

## The *Original* Version of ARM: a Pioneer Technology for Rhodopsin QM/MM Modeling

### State-of-the-Art for QM/MM Modeling of Rhodopsins

The past few decades have witnessed a growing interest in developing hybrid $\text{QM/MM}$ approaches to tackle problems in computational photochemistry and photobiology [27]. A remarkable advantage of using hybrid $\text{QM/MM}$ methodologies is that in silico models featuring a high-level of complexity can be properly constructed, through the definition of subsystems, each treated at a different level of theory (or layers) according to the required level of accuracy.

**Fig. 5:**
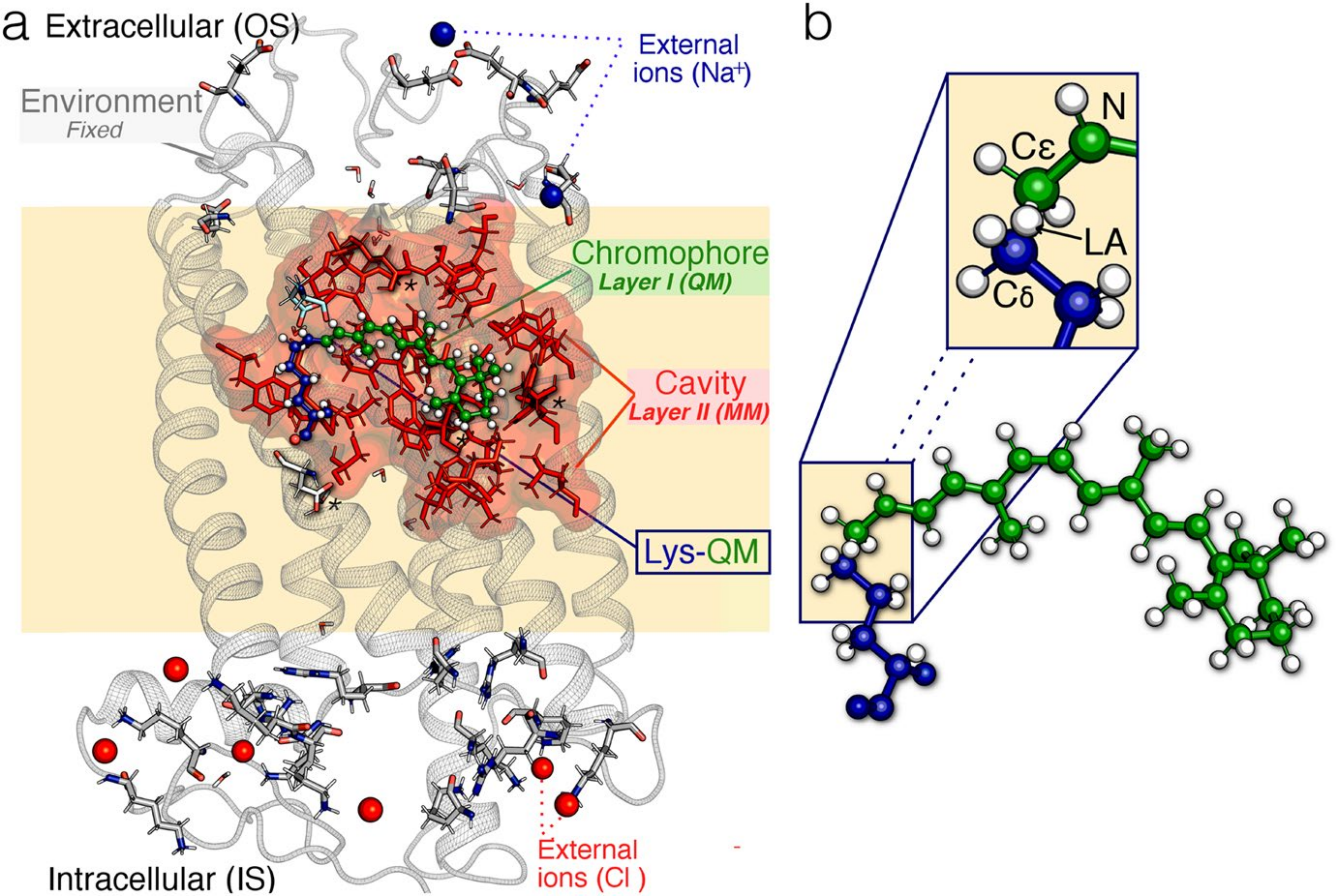
General structure of a monomeric, gas-phase and globally uncharged Automatic Rhodopsin Modeling (ARM) quantum-mechanics molecular mechanics (QM/MM) model. **a** Relationship between the ARM model’s three subsystems and two multiscale layers. Detailed description of the components of the three subsystems (for an animal rhodopsin from the DA state of Bovine rhodopsin). Gray cartoon Environment subsystem, green ball-and-sticks $r\text{PSB}$ chromophore, blue ball-and-sticks lysine side-chain covalently linked to the $r\text{PSB}$ chromophore, cyan tubes main chromophore counterion, tubes marked with * residues with non-standard protonation states, red spheres external Cl$^{-}$ , blue spheres Na$^{+}$ counterions, red/white tubes crystallographic water molecules, red frames surface amino acid residues forming the chromophore cavity subsystem, gray tubes external OS and IS charged residues. **b** The $r\text{PSB}$ chromophore (green) and the linked Lys side-chain fragment (blue) form the Lys-QM subsystem, which includes the H-link atom located along C$\varepsilon$–C$\delta$ connecting blue and green atoms (inset:* LA* H-linked atom), which belong to the $\text{MM}$ and $\text{QM}$ parts of the model. Adapted with permission from [40]. Copyright 2019 American Chemical Society

As shown in Fig. 5 and further described in detail in Ref. [43], a basic $\text{QM/MM}$ model for a biological photoreceptor (e.g., rhodopsin) should feature at least three subsystems: (1) the reactive part of the system, or prosthetic group, that carries the photochemical process (i.e., chromophore), treated with a suitable, and usually computationally expensive, $\text{QM}$ method (see Section 2.1.1 in Ref. [43]); (2) the residues that, due to either steric or electrostatic interactions with the prosthetic group, directly influence the role of the reactive part (i.e., amino acids and water molecules forming the chromophore cavity), treated classically with a less expensive (optionally polarizable), $\text{MM}$ force field (see Section 2.1.2 in Ref. [43]); and (3) the residues without an evident role on the photochemical process (i.e., protein environment) structurally fixed during the calculation and treated as point charges (see Fig. 5). Usually, $\text{QM/MM}$ models are complex and, unfortunately, not univocally defined, thus Ref. [43] collates the different approaches to modeling the photochemical properties of $\text{Bovine rhodopsin from } Bos\,taurus \text{ (Rh)}$, a rhodopsin for which the X-ray structure is available [2, 53, 54] and that, also for this reason, is often taken as a reference for the benchmarking of different $\text{QM/MM}$ models. Differences in construction protocols of the $\text{QM/MM}$ setup lead to variations in computed $\lambda _{\max }^{a,\text {calc}}$ up to 41 nm [29–31, 55–58].

### ARM Scope

It is important to define a standardized protocol for the fast and automated production of congruous $\text{QM/MM}$ models, which can subsequently be replicated in any laboratory. Such a protocol should not strictly aim at the prediction of the absolute values of observable properties, but to the description of their changes along sets of different rhodopsin variants. The ARM protocol described in this work represents our attempt to provide such a tool. The protocol is based on two well-defined phases called “generators”: the (1) input file generator and (2) QM/MM model generator. Sections 3.3 and 3.4 describe the development of the original version of the $\text{ARM}$ protocol [59], which provided the QM/MM model generator. Furthermore, Ref. [59] provides a series of instructions for the manual preparation of the input file, which served for the subsequent development of the input file generator presented in Sect. 4.

The original $\text{ARM}$ protocol is designed for a semi-automatic, fast and parallel building of congruous sets of $\text{QM/MM}$ models of wild-type and mutant rhodopsin-like photoreceptors [59]. Accordingly, as illustrated in Fig. 5, this version provides specialized $\text{QM/MM}$ models that, in general, would not be applicable to other (e.g., cytoplasmic) photoresponsive proteins, or even to rhodopsins that contain artificial retinal chromophores. In the general framework of the QM/MM model generator, one needs to consider (1) the wise division of the complex molecular system into different, simpler subsystems, and (2) the definition of particular layers that represent the approaches (i.e., levels of theory) used for the proper description of each of the subsystems.

To assess point (1), one can refer to Sect. 2.1, where it is specified that rhodopsins belonging to the three known families (i.e., animal, microbial and heliorhodopsins) share a common architecture constituted by a protein environment featuring seven transmembrane helices, which form a cavity hosting the retinal chromophore (see Fig. 5a). As illustrated in Fig. 5b, an important feature to be considered is that the chromophore is linked covalently via a specific lysine residue (e.g., located in the middle of helix VII and helix G for animal and microbial rhodopsins, respectively), via an imine (–C=N–) linkage, forming the $r\text{PSB}$.

To evaluate point (2), instead, it is crucial to identify the chemical/physical phenomena to be modeled and the target properties to be computed. Section 2.3 shows that the process driving the diverse biological functions of rhodopsins is the photoisomerization of the $r\text{PSB}$ chromophore, occurring immediately after the absorption of a photon of the appropriate wavelength. As illustrated in Fig. 4, the most relevant properties to be reproduced/predicted are the rhodopsin color (or absorption wavelength), fluorescence, and photochemical reactivity.

The scope of ARM, since inception, has been that of being capable of reproducing experimental trends in rhodopsin series. In other words, it was not designed to be a predictive, but rather an investigative, tool; given a set of rhodopsins, the generated corresponding models should reflect trends in spectroscopic and/or photochemical properties. In turn, the computed models could be used to investigate, at the molecular level, the origin of such property changes.

### Definition of an ARM QM/MM Model

As previously mentioned, it is possible to subdivide the rhodopsin structure into three subsystems. Figure 5a exemplifies how these subsystems are defined, using the case of $\text{Rh}$ rhodopsin: the (protein) environment (gray cartoon), the chromophore cavity (red frames/surface), and the Lys-chromophore (blue/green ball-and-sticks). The protein environment sub-system features residues (backbone and side-chain atoms) fixed at the crystallographic or comparative (homology) structure, and incorporates external Cl$^{-}$ and/or Na$^{+}$ counterions (see discussion below) also fixed at pre-optimized positions. The chromophore cavity sub-system, instead, contains residues with fixed backbone and relaxed side-chains. The Lys-QM system contains the atoms of the covalently linked lysine side-chain in contact (through C$\delta$) with the $\text{QM/MM}$ frontier and the entire $\text{QM}$ subsystem, which corresponds to a *N*-methylated retinal chromophore. All the Lys-QM atoms are free to relax during the $\text{QM/MM}$ calculation.

Accordingly, the $\text{ARM QM/MM models}$ illustrated in Fig. 5 can be defined as basic, monomeric, gas-phase and globally uncharged, with electrostatic embedding. The term “gas-phase” refers to the fact that, although rhodopsins are proteins exposed to trans-membrane electrostatic fields (i.e., few tenths of meV) occasioned by an asymmetric distribution of the surface ions, in the $\text{ARM}$ basic approach, the interactions between the protein and the membrane, as well as solvation effects, are not modeled explicitly. Instead, the protocol mimics the situation of a globally uncharged protein by adding external Cl$^{-}$ and/or Na$^{+}$ counterions, properly placed near the most positively and/or negatively charged surface amino acids, in both the intracellular ($\text{IS}$) and extracellular ($\text{OS}$) protein surfaces.

**Fig. 6:**
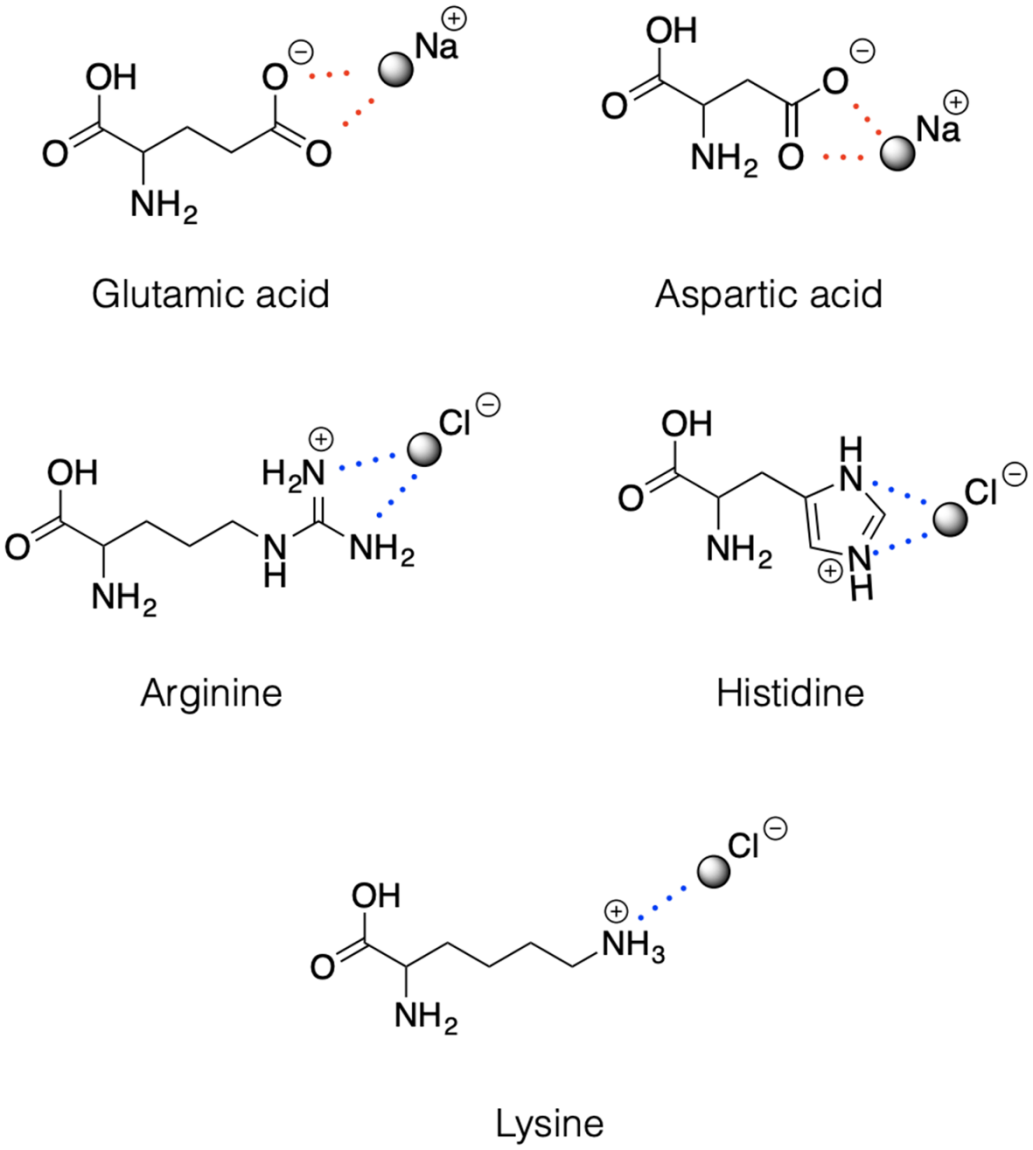
External counterion positions relative to ionizable residues, according to the No Surface Charge (NSC) scheme used for the ARM QM/MM models Schematic representation of the positions of the external counterions (i.e., Na$^{+}$ and Cl$^{-}$) used to neutralize the $\text{IS}$ and $\text{OS}$ surfaces in the gas-phase $\text{ARM QM/MM models}$. Reproduced with permission from [59] Copyright 2016 American Chemical Society

To this aim, $\text{ARM}$ uses the No (net) Surface Charge (NSC) scheme in which the user must execute the following operations manually: (1) identify the number of positively and negatively charged surface residues, (2) calculate independently the charge in the $\text{IS}$ and $\text{OS}$ , (3) determine the number of Cl$^{-}$ and/or Na$^{+}$ to be added, and (4) position the external counterions as illustrated in Fig. 6. As described in Sect. 4, in the most updated version of the protocol, such a procedure is performed automatically by a specific algorithm. Moreover, to account for the water-mediated hydrogen-bond network (HBN) in the protein cavity, the internal crystallographic water molecules are retained in the model, while the water molecules that are not detected experimentally are assumed to be extremely mobile or just absent in the chromophore hydrophobic protein cavity.

### QM/MM Model Generator

**Fig. 7:**
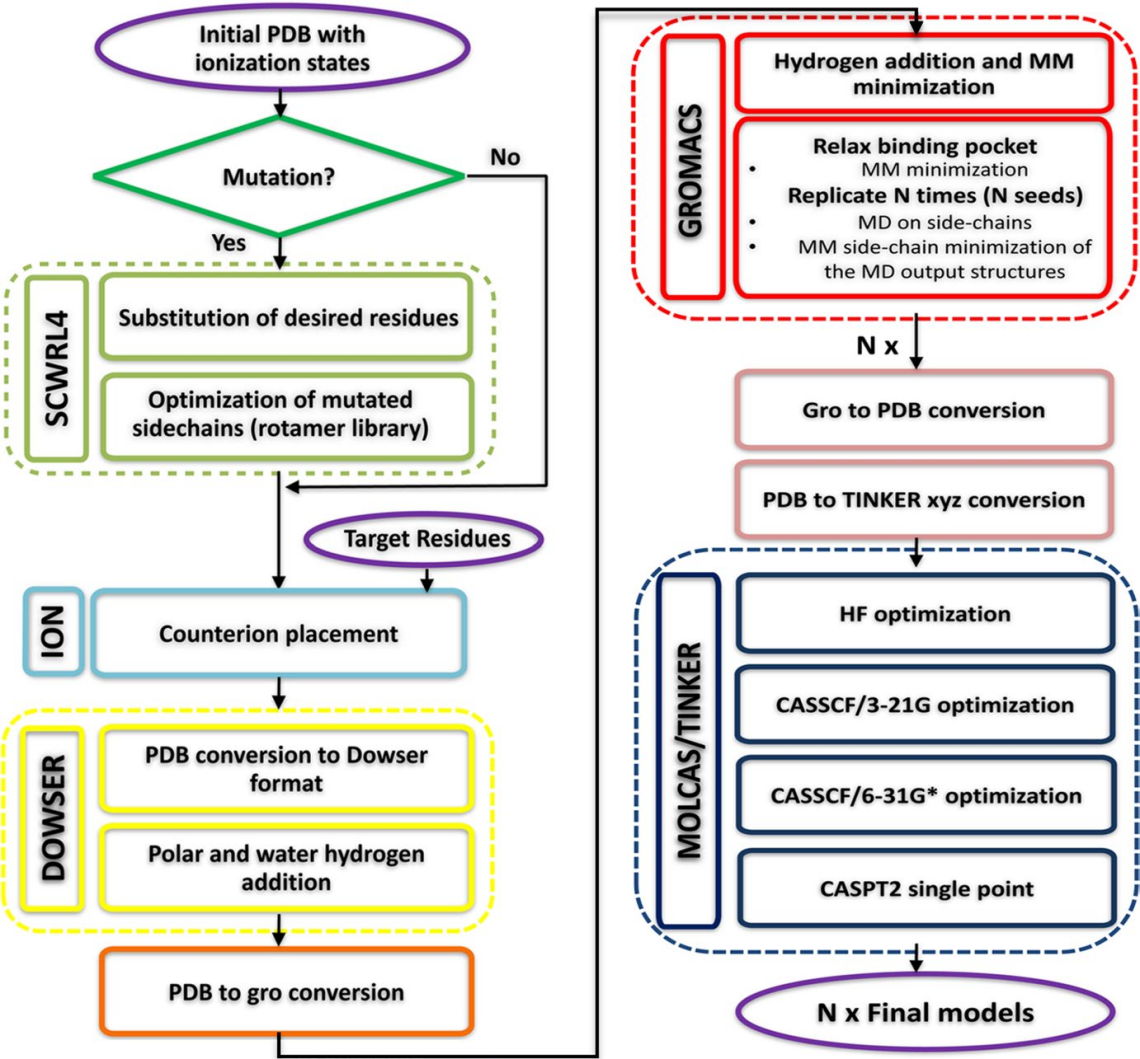
General workflow of the QM/MM model generator developed in the original version of ARM. The procedure starts with the previously prepared input structure and finishes with the $\text {S}_{0}$ $\text{ARM QM/MM model}$, that consists on 10 replicas of the optimized structure along with the computed $\text{Maximum absorption wavelength}$ ($\lambda _{\max }^{a}$). The different steps of the protocol, as well as the level of theory and the used software are provided Adapted with permission from [59]. Copyright 2016 American Chemical Society

Figure 7 illustrates the general workflow of the QM/MM model generator proposed in Ref. [59] for the semi-automatic building of ground-state $\text{ARM QM/MM models}$ of rhodopsins and subsequent computation of the $\text{Maximum absorption wavelength}$, via vertical excitation energy calculations.

#### Classical Molecular Dynamics Simulations

A preliminary preparation of the input structure is required, which consists of the following steps:

1. Selection and optimization (i.e., energy criteria) of all the water molecules, using the crystallographic/comparative positions as initial guess.
2. Addition of hydrogen atoms to polar residues and waters.
3. Addition of hydrogen atoms to the other residues of the protein environment, chromophore cavity and chromophore subsystems.
4. $\text{MM}$ energy minimization on the added hydrogen atoms.
5. $\text{MM}$ geometry optimization on all the side-chains of the residues of the chromophore cavity subsystem.
6. Generation of *N*=10 independent models (replicas) to simulate and explore the possible relative conformational phase space of the cavity residue side-chains and retinal chromophore.

Steps 1 and 2 are performed using the program DOWSER [60]. These steps ensure proper treatment of water and $\text{Hydrogen-bond networks (HBN)}$, which affect side-chain conformations and long-range electrostatics, thus ultimately modifying spectral and photochemical properties.

Steps 3–6 are performed using the program GROMACS [61]. Step 6 uses classical molecular dynamics simulations ($\text{MD}$) to perform a simulated annealing relaxation at 298 K on all side-chains of the Lys-QM and cavity subsystem, keeping the backbone fixed at the crystallographic/comparative structure; during the $\text{MD}$ computation the retinal chromophore subsystem is also allowed to move. To warrant independent initial conditions, each of the *N*=10 independent $\text{MD}$s starts with a different, randomly chosen seed. Note that, during the MD run, the chromophore subsystem is represented using an $\text{MM}$ parameterization and partial charges computed as AMBER-like $\text{Restrained Electrostatic Potential (RESP)}$ charges, which are specific for each used isomer of the chromophore (e.g., 11-*cis*, all-*trans* and 13-*cis* $r\text{PSB}$). The corresponding parameterized RESP point charges, currently used in $\text{ARM}$, are reported in the Supplementary Information of Ref. [59]). The default heating, equilibration, and production times for the $\text{MD}$ (selected via benchmark calculations in Ref. [59]) are 50, 150, and 800 ps, respectively, for a total length of 1 ns. In each run, the *frame closest to the average* of the 1 ns simulation is then selected as the starting geometry (i.e., guess structure) for constructing the corresponding $\text{QM/MM}$ model. Melaccio et al. [59] have shown that, for a set of three phylogenetically diverse rhodopsins ($\text{Rh}$, $\text{SqRh}$ and $\text{ASR}$ $_{13C}$), *N*=10 replicas are enough to provide sufficient variability.

#### QM/MM Calculations

As shown in Fig. 7, each of the 10 replicas generated as guess structures undergoes a series of $\text{QM/MM}$ calculations, defining a well-established protocol. In this regard, Fig. 5b illustrates the atoms that are considered as the Lys-QM layer during these calculations, corresponding to the full N-methyl retinal chromophore (*i*.*e*, $\text{QM}$ subsystem; 53 atoms) and its covalently linked lysine side-chain of the $\text{MM}$ subsystem (i.e., 9 atoms). The $\text{QM/MM}$ frontier is treated within a link atom approach (see Fig. 5b), whose position is restrained according to the Morokuma scheme, and is placed across the lysine C$\delta$–C$\varepsilon$ bond (where C$\varepsilon$ is a $\text{QM}$ atom). The lysine charges are modified by setting the C$\delta$ charge to zero to avoid hyperpolarization and to redistribute the residual fractional charge on the most electronegative atoms of the lysine, thus ensuring a +1 integer charge of the Lys-QM layer.

The following $\text{QM/MM}$ calculations are performed sequentially, using the program [Open]Molcas/Tinker [62–64]:

1. Geometry optimization at the HF/AMBER/3-21G level.
2. Geometry optimization at the 2-roots single-state CASSCF(12,12)/AMBER/3-21G level.
3. Geometry optimization at the 2-roots single-state CASSCF(12,12)/AMBER/6-31G(d) level.
4. Inclusion of the electron correlation via a single point energy calculation at the 3-roots state-average CASPT2(12,12)/6-31G(d) level.

The sequential optimizations steps 1–3 aim at a more rapid convergence of both the molecular orbitals and geometry, and use “microiterations” that provide quicker convergence, lower energies, and a more realistic description of chromophore-environment interactions [65]. Besides, suitable level shifting values are used during the CASSCF and CASPT2 calculations, to minimize the possibility of convergence failure due to state mixing and intruder state problems. The CASPT2//CASSCF/6-31G(d)/MM treatment [55] has been investigated extensively for photobiological studies and its limitations are well understood. As previously documented [59], the rather small (ca. 3–4 kcal mol$^{-1}$) error in excitation energy reported in several studies for this level of theory, is somewhat due to error cancellations associated with the limited quality of single-state CASSCF/AMBER/6-31G(d) equilibrium geometries. Therefore, the different properties computed by $\text{ARM}$ are expected to be affected by systematic error cancellations. Nevertheless, the main focus of $\text{ARM}$ is the ability to reproduce observed trends in vertical excitation energies (i.e., the sign and magnitude of the individual differences concerning experimental data).

As observed in Fig. 7, the final output consists of 10 replicas of equilibrated $\text{QM/MM}$ models of the type described in Sect. 3.3 and, for each replica, the vertical excitation energy values between $\text {S}_{0}$ and the first two singlet excited states $\text {S}_{1}$ and $\text {S}_{2}$ is provided.

### Automation Issues

The *original* $\text{ARM}$ protocol provides basic, gas-phase and computationally fast $\text{QM/MM}$ models for comparative studies, to predict photochemical property trends (e.g., for large arrays of rhodopsin variants) that fit selected sets of experimental data (mainly $\lambda _{\max }^{a}$ values), within a well-established error bar. However, the general target in the formulation of an automated modeling of rhodopsins is to achieve a protocol featuring the following features for both the input file generator and QM/MM model generator phases:

1. **transferability**, so as to properly describe rhodopsins with differences in protein sequence (i.e., organism belonging to different life domains and kingdoms; see Sect. 2.1), and different configurations of the $r\text{PSB}$;
2. **documented accuracy**, so as to be able to translate results obtained in silico into hypothesis that can be proved experimentally;
3. **reproducibility**, so as to be reproduced in any laboratory starting the model building from scratch;
4. **speed** and **parallelization**, so as to achieve the fast generation of large arrays of rhodopsins variants (i.e., wild-type and mutants) simultaneously;
5. **automation**, so as to reduce building errors (i.e., human factors) and avoid biased $\text{QM/MM}$ modeling.

In order to assess whether or not the original $\text{ARM}$ satisfies each of the points (1)–(5) described above, the protocol was benchmarked on the prediction of trends in $\lambda _{\max }^{a}$ for a limited set of 10 wild-type and 17 mutant rhodopsins [59]. Accordingly, points (1) and (2) were successfully achieved, while points (3), (4) and (5) were accomplished only partially. More specifically, point (1) was achieved since the predicted trends in $\lambda _{\max }^{a}$ presented a good agreement with experimental data (i.e., error bar of ca. 4.0 kcal mol^−1^). Point (2) has been recently demonstrated via collaborative experimental and computational studies attempting enhancement of either color tuning [42] and fluorescence [66] properties for microbial rhodopsins, achieving novel applications in optogenetics. Further applications can be found in [43]. The main drawback of the original $\text{ARM}$ is that it does not include a computational tool for the automatic, or even semi-automatic, generation of the input files (i.e., no input file generator). Input file generation is, instead, achieved through a manual manipulation of the template structure [59]. Such an input file is based on a X-ray crystallographic structure or comparative model of the protein in PDB (Protein Data Bank) format [67, 68], which contains the information specified in the caption of Fig. 5 and summarized as follows:

(i) the selected monomeric chain structure, including the $r\text{PSB}$ chromophore, crystallographic/comparative water molecules, and excluding membrane lipids and non functional ions;
(ii) a list of residues forming the chromophore cavity;
(iii) the protonation states for all the internal and surface ionizable amino acid residues;
(iv) suitable external counterions (Cl$^-$/Na$^+$) needed to neutralize both IS and OS protein surfaces.

Due to possible different user choices (e.g., during the placement of $\text{IS}$ and $\text{OS}$ counter-ions; see Fig. 5a), reproducibility of the results described (point (3)) cannot be guaranteed. In addition, the input preparation required a few hour user’s manipulation of the template protein structure (i.e., a skilled user completes the preparation of an ARM input for a new rhodopsin in not less than 3 h and after taking a series of decisions based on their chemical/physical knowledge and intuition). Such limitations, added to the human error factor, represent a serious issue when the target is the generation of hundreds rhodopsin models. Therefore, due to manual interventions of the user in the input generation, speed and parallelization (point (4)) are not guaranteed. Of course, the lacking of an automatic input file generator and the current methodological issues of the QM/MM model generator described below, made the protocol semi-automatic rather than automatic (i.e., also point (5) was not accomplished).

Additionally, as specified by Melaccio et al. [59], the code was written as a series of independent bash-shell scripts that are not interconnected by a general driver. Therefore, as explained in the Supporting Information in [59], for each step of the protocol, the user should execute each script manually and make a series of choices via a command-line assisted tool. This features the QM/MM model generator as a semi-automatic rather than an automatic tool. The following sections will show how each of these problems have been overcome.

## *a*-ARM: the First Major Update Towards Automation

(Most of the content of the following four sections is reproduced/adapted with permission from [69], copyright 2019 American Chemical Society, while content of Sect. 4.5 is reproduced/adapted with permission from [35], open access under a CC BY license (Creative Commons Attribution 4.0 International License)).

### Methodological Aspects

**Fig. 8:**
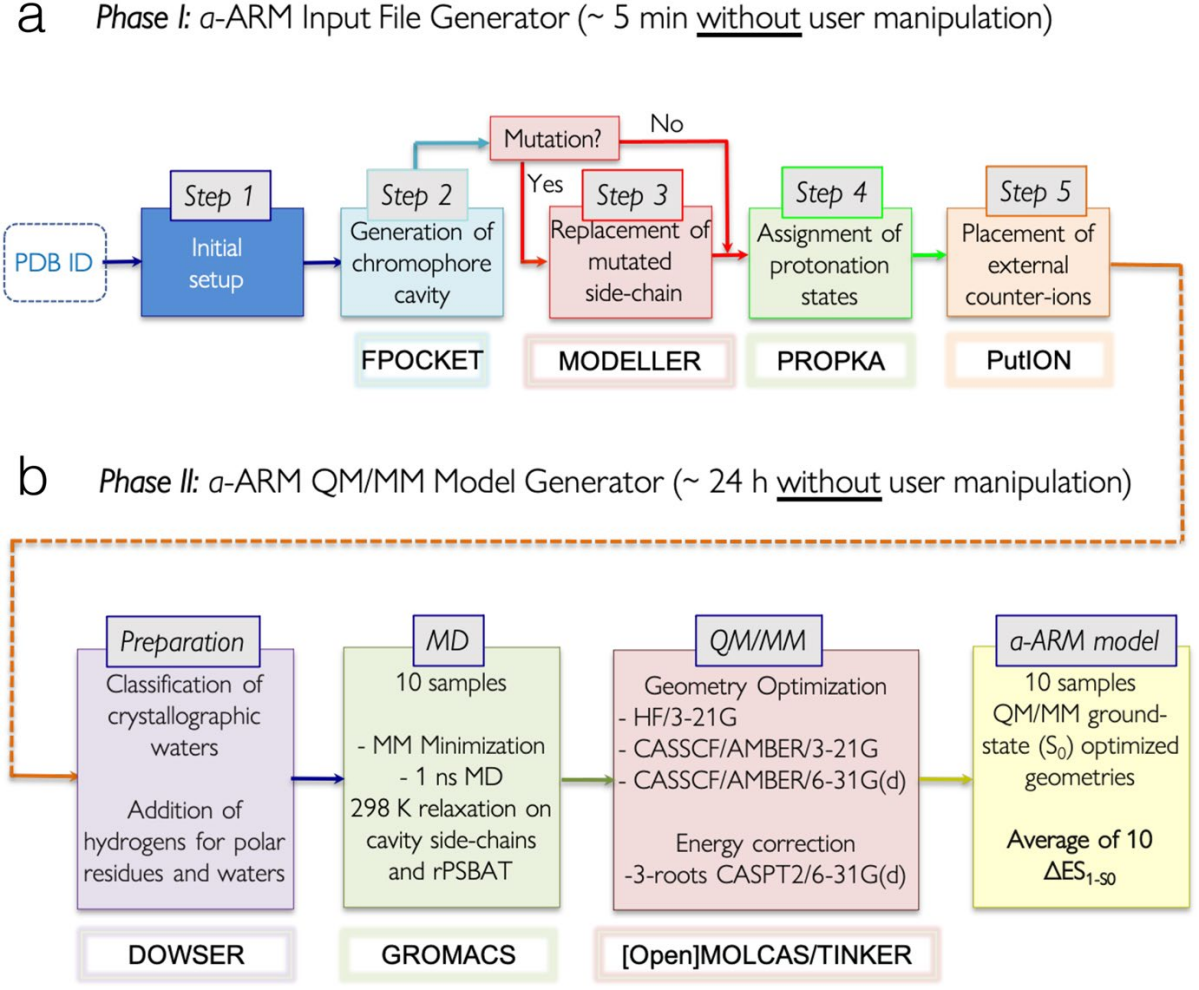
General workflow of the two phases of the ${a}\text {-}$ARM rhodopsin model building protocol. **a** Input file generator phase. **b** QM/MM model generator phase

This section presents the *advanced* ${a}\text {-ARM}$ [69], an updated version of the *original* semi-automatic $\text{ARM}$ protocol [59] (Sect. 3) that, as main novelty, features an input file generator phase for either the fully automatic or semi-automatic computer-aided building of the $\text{ARM input}$. This updated version overcomes most of the automation issues of the original $\text{ARM}$, highlighted in Sect. 3.5, by including methodological improvements that lead to more reliable and reproducible $\text{QM/MM}$ models.

The first aim of the $a$-ARM model building is to be consistent (in terms of output) with the original protocol, as described in Sect. 3.3 and shown in Fig. 5. Figure 8 shows the general workflow of ${a}\text {-ARM}$, which encapsulates two well-defined and automated phases (panels a and b), hereafter referred to as Phase I and Phase II, respectively. Whereas the latter is substantially the same QM/MM model generator phase reviewed in Sect. 3.3 (i.e., in terms of methodology, although not of implementation), the former is the new input file generator phase.

**Fig. 9:**
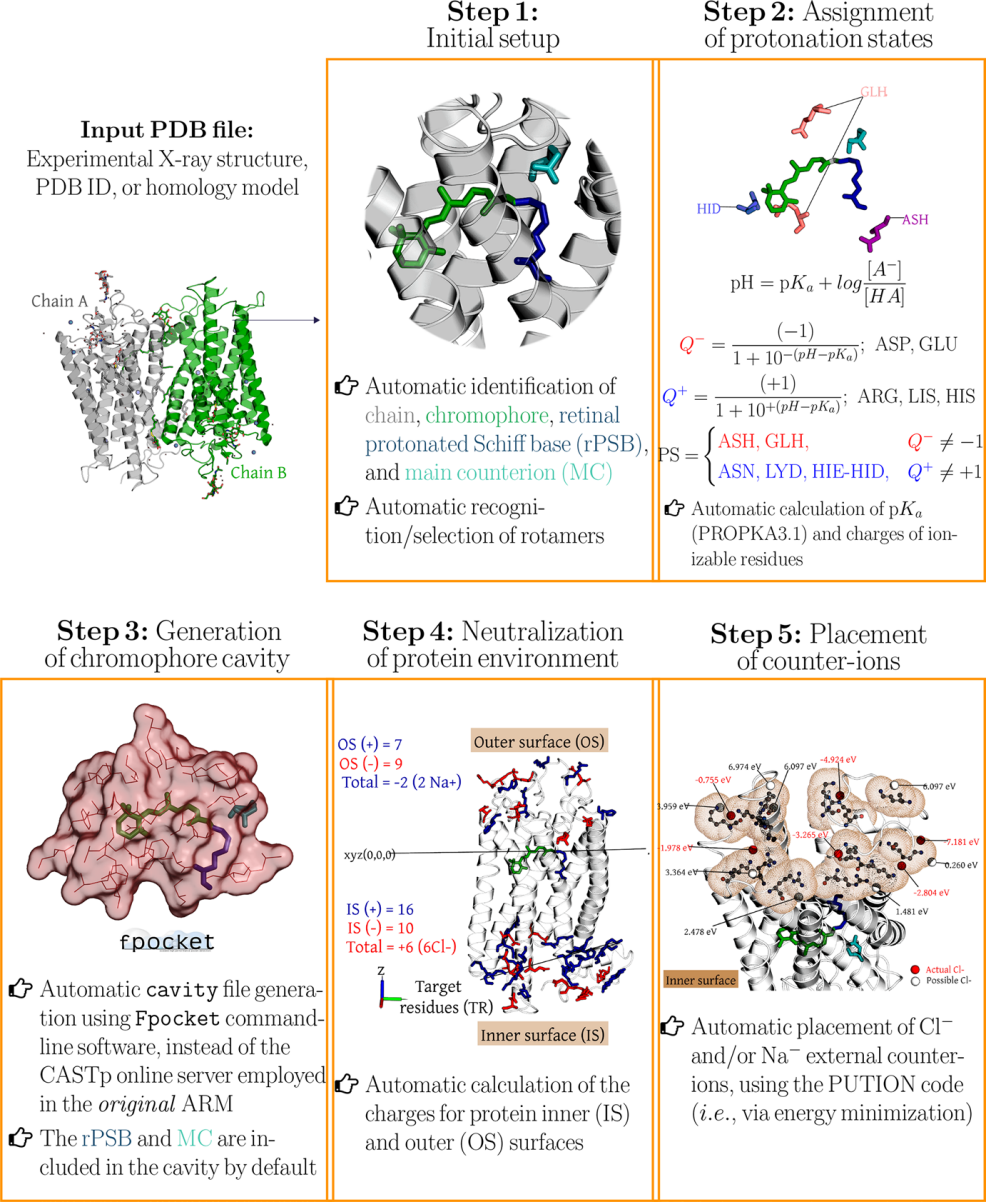
Overview of the most relevant features of the input file generator, introduced in the ${a}\text {-ARM}$ version of the protocol. Methodological and automation improvements achieved with the input file generator, in terms of: initial setup; automatic strategy adopted for the assignment of protonation states for ionizable residues; replacement of the software (i.e., fpocket instead of CASTp) for the automatic generation of the chromophore cavity; automatic approach to define the charge of the $\text{IS}$ and $\text{OS}$ surfaces and automatic counterion placement based on energy minimization

The combined use of the two phases achieves the automatic building of $\text{Ground-state}$ $\text{ARM QM/MM model}$s, starting from the structure of a rhodopsin in PDB format, either as PDB code or as a comparative homology model. As observed in Fig. 8, the initial structure is processed by Phase I to obtain the $\text{ARM input}$, which is subsequently processed by Phase II to obtain the $\text{ARM QM/MM model}$ (i.e., gas-phase equilibrated optimized $\text {S}_{0}$ structure) and the predicted average $\lambda _{\max }^{a}$. As further explained in Ref. [69], the input file generator is implemented as a user-friendly command-line interface, where the researcher interacts with the program by typing information directly in the computer terminal, without the need to manipulate text files or visualize chemical structures, as previously necessary in the original “manual” strategy. Refs. [69] (see Section S1 in the SI) and [43] (see Section 3.1 in this reference) report a detailed description of both manual (i.e., *original* $\text{ARM}$) and automatic procedures used to pursue steps 1–5 of Fig. 8a, with particular emphasis on the improvements achieved with ${a}\text {-ARM}$, as well as in its higher level of automation. Figure 9 presents an overview of such improvements.

One of the most remarkable features of the new protocol is that, given the options (i.e., parameters) selected in steps 1–5 of phase I (Fig. 8a), ${a}\text {-ARM}$ allows either the automatic or semi-automatic computer-aided production of the $\text{ARM input}$. Accordingly, ${a}\text {-ARM}$ is sub-divided in ${a}\text {-ARM}$_default_ (see Section 3.1 of Ref. [69]) and ${a}\text {-ARM}$_customized_ (see Section 3.2 of Ref. [69]) approaches. The former refers to a fully automatic input generation, which uses default parameters as suggested by the code (i.e., chain, rotamers or side-chain conformations, pH, protonation states, residues forming the chromophore cavity), whereas the latter allows the computer-aided customization of some of such parameters when the default choices are not suitable.

**Fig. 10:**
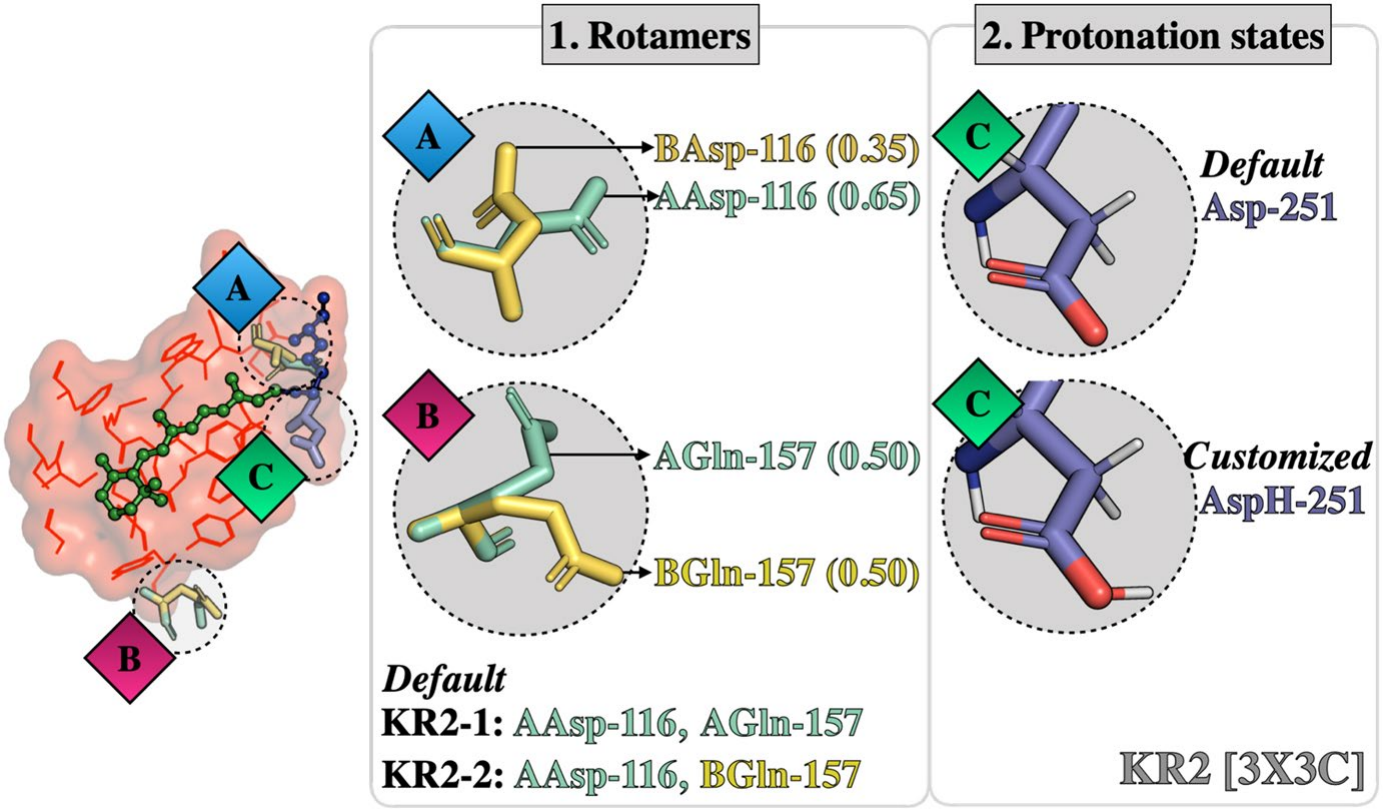
Default and customized *a*-ARM models for Krokinobacter rhodopsin 2 from *Krokinobacter eikastus* (KR2) [PDB ID 3X3C [70]]. Left: conformational (the occupancy factor of the rotamers Asp-116 and Gln-157 are presented in parentheses). Right: ionization state variability

**Fig. 11:**
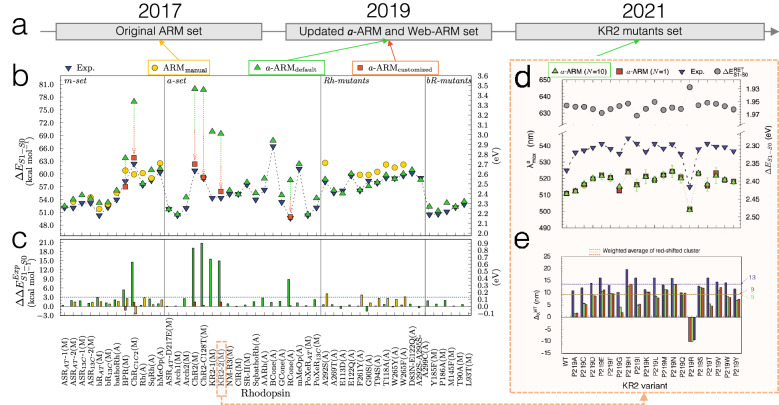
Evolution and benchmarking of the ARM protocol over time, in terms of reproduction of experimental trends in $\lambda _{\max }^{a}$. **a** Timeline for the benchmark sets used for testing each version of ARM over the years. **b** Comparison between vertical excitation energies ($\Delta E_{S1-S0}$) computed with either *a*-ARM$_{{\text {default}}}$ (up green triangles) or *a*-ARM$_{\text{ customized}}$ (red squares) [69], and ARM$_{\text {original}}$ [59] (yellow circles) and experimental data (down blue triangles), along with the **c** differences between computed and experimental $\Delta E_\text {S1-S0}$ ($\Delta \Delta E_\text {S1-S0}^{Exp}$). The *m*-set corresponds to wild-type (WT) rhodopsins forming the original benchmark set for the ARM protocol; *a*-set introduces new rhodopsins to the benchmark set of the *a*-ARM protocol; $Rh-mutants$ set contains mutants of bovine Rhodopsin (Rh) belonging either to the benchmark set of the ARM or *a*-ARM protocols (see Refs. [59, 69]); and $bR-mutants$ set contains mutants of bR evaluated with the Web-ARM interface (see Sect. 5 and Ref. [74]). **d** Comparison between $\Delta E_{S1-S0}$ computed with ${a}\text {-ARM}$, average value (up green triangles) and representative seed (red squares), and experimental data (down blue triangles) for WT and P219X mutants of KR2 [6REW], along with **e** the differences between these values for each mutant with respect to the WT (see Ref. [35]). Adapted with permission from [69]. Copyright 2019 American Chemical Society and [35], open access under a CC BY license (Creative Commons Attribution 4.0 International License)

The customized approach is used in cases where the default choices produce outlying models that fail to reproduce trends in absorption properties. Several examples are presented in Refs. [35, 43, 69]. For instance, Fig. 10 illustrates how the customization, in terms of either selection of side-chain conformations and protonation states, is achieved for the case of the microbial rhodopsin $\text{Krokinobacter rhodopsin 2 from} \,{Krokinobacter \,eikastus} \,\text{(KR2)}$ [35, 69, 70]. As observed, in the 3X3C X-ray structure [70], the residue Asp-116, considered as the main counterion (MC) of the $r\text{PSB}$, exhibits two side-chain conformations, namely, AAsp and BAsp, labeled with occupancy numbers 0.65 and 0.35, respectively. Moreover, the residue Gln-157 that is part of the environment subsystem (i.e., fixed during the $\text{QM/MM}$ calculations) presents two conformations (AGln and BGln) both with 0.5 occupancy. According to the occupancy numbers, ${a}\text {-ARM}$_default_ selects the rotamer AAsp-116 and generates two models relative to Gln-157: KR2-1, which includes AAsp-116 and AGln-157, and KR2-2, which includes AAsp-116 and BGln-157. The computed $\Delta \text {E}_{S1-S0}^{a}$ for both default models, presented below in Fig. 11, features an error of about 15.0 kcal mol$^{-1}$ with respect to experimental data. Since the default models are unable to provide values inside the experimental trend, the ${a}\text {-ARM}$_customized_ approach is necessary. As shown in the right panel of Fig. 10, such customization is performed through a more rational assignment of the protonation states of the two aspartic acid residues forming the counterion complex of the $r\text{PSB}$, namely Asp-116 and Asp-251 [35]. The default model predicts that both aspartic acids are negatively charged. However, as further discussed in Refs. [35, 43, 69], the presence of these two negative charges would outbalance the single positive charge of the $r\text{PSB}$, generating the large blue-shifted effect mentioned above. Accordingly, in the customized model the secondary counterion (SC) Asp-251 is, instead, protonated (i.e., neutral) to counterbalance the charge in the vicinity of the $r\text{PSB}$. As can be seen in Fig. 11, such customization provides a model with a small error bar of about 1.5 kcal mol$^{-1}$. An updated $\text{ARM}$ model of KR2 was recently reported [35]. Although such a model was constructed starting from a different template X-ray structure [PDB ID 6REW [1]], the same customized setup for the protonation states of the counterion complex of the $r\text{PSB}$ (i.e., deprotonated Asp-116, protonated Asp-251) was found. Remarkably, such ${a}\text {-ARM}$ $_\text {customized}$ model was used as a starting structure for modeling a set of 19 mutants and for reproducing not only experimental trends in $\Delta \text {E}_{S1-S0}^{a}$, but also giving further insights into the mechanism of color tuning in the position Pro-219 of KR2 [35].

Notice that in the ${a}\text {-ARM}$ benchmark set (Fig. 11) a similar large blue-shifting has been observed in $\Delta \text {E}_{S1-S0}^{a}$ for all ${a}\text {-ARM}$_default_ models that predict two negative charges near the $r\text{PSB}$ (see Tables S2 and S3 in Ref. [69]). The customization procedure (see below) to obtain a $\Delta \text {E}_{S1-S0}^{a}$ within the error bar of the protocol, always implies the neutralization of one of these two charges. This is, in fact, one particularity of the simplified structure/scheme of the ${a}\text {-ARM}$ models.

In general, protonation state assignment for ionizable residues remains a basic issue for current $\text{QM/MM}$ protein modeling. No robust method is available that guarantees a correct choice of a pKa value, due to the complexity of the protein environments and its interconnected local effects. Furthermore, a residue may be present as an equilibrium between ionized and not-ionized forms, hence carrying only a partial charge. These issues are a current research matter [35, 71–73].

Despite the difficulties mentioned above, ${a}\text {-ARM}$ adopts the following guiding customization procedure. As shown in the latter example, customized $\text{ARM QM/MM model}$s can be constructed according to well-defined operations that can be easily replicated. Ref. [69] proposes to focus on the selection of the ionization states and side-chain conformations only. Accordingly, the customization of the protonation states involves three phases: (1) at pH > 6 the ionization states are modified by setting the pH to 5.2 in step 4 (see Fig. 8b); (2) the protonation state of the main and secondary counterions of the rPSB are checked, and if the analysis shows them both ionized the secondary counterion is neutralized; (3) in case the model generated in step (2) does not reproduce the experimental absorption maxima, then the secondary and main counterion ionization states are exchanged (see also the Supporting Information of Ref. [35] for further details on, e.g., the pH value choice). Note that in QM/MM modeling, it is a common practice to evaluate the protonation states of the $r\text{PSB}$ counterion complex by looking, as a guide, at the reproducibility of the experimental $\lambda _{\max }^{a}$ (see, for instance, Refs. [35, 71–73])

Indeed, the novelty of the default and customized approaches is that, regardless of the user or computational facility, reproducible inputs, and consequently reproducible $\text{ARM QM/MM model}$s, are guaranteed when the same parameters are used. This represents an advancement with respect to the original version since it allows for the models to be reproduced in any laboratory and by any user, even when starting building an $\text{ARM QM/MM model}$ from scratch (see point (3) of Sect. 3.5).

### Software Implementation Aspects

The computational implementation of both the input file generator and the QM/MM model generator phases as Python-based, modular codes boosted the building of the PyARM software package that will be introduced in Sect. 6. Moreover, their ease of transferability, allowed their use behind the Web-ARM web page, which will be described in Sect. 5.

Furthermore, although ${a}\text {-ARM}$ can presently build only rhodopsin models (i.e., with natural retinal), it provides a template for the development and generation of an automatic $\text{QM/MM}$ building strategy for other, more general, systems such as rhodopsins incorporating artificial (i.e., unnatural) chromophores. This is straightforwardly achieved given the modular architecture of the PyARM package and the fact that any chromophore can be treated when using the appropriate force field.

Finally, it is worth stressing that the new protocol achieves all the features (1)–(5) described in Sect. 3.5, overcoming the automation limits of the original version.

### Benchmark, Validation and Application Aspects

Figure 11 shows the current validation of the ${a}\text {-ARM}$ protocol through the prediction of trends in $\lambda _{\max }^{a}$, performed using a benchmark set of 44 animal and microbial rhodopsin variants (i.e., 25 wild type and 19 mutants) that come from different organism and are phylogenetically diverse [69, 74] (see Fig. 11b). The full benchmark set features values ranging from 458 nm (62.4 kcal mol$^{-1}$, 2.71 eV) to 575 nm (49.7 kcal mol$^{-1}$, 2.15 eV). Such a relatively wide range provides information on the method accuracy, while the rhodopsin diversity provides information on the transferability and general applicability of the generated models. Figure 11b, c are divided in four different regions: *m*-set, *a*-set, $Rh-mutants$ set, and $bR-mutants$ set. The *m*-set and $Rh-mutants$ set are used to compare the performance of original $\text{ARM}$ and ${a}\text {-ARM}$ versions, while the remaining sets focus exclusively on the performance of *a*-ARM.

The ${a}\text {-ARM}$_default_ approach proved to be capable of reproducing the $\Delta \text {E}_{S1-S0}^{a}$ values for 86% of cases (38/44), with an error lower than 4.0 kcal mol$^{-1}$ (0.13 eV), whereas the other 14% cases were successfully obtained with the ${a}\text {-ARM}$_customized_ approach (i.e., changing the side-chain conformation and/or protonation states pattern). A detailed description of the customization procedure used for reproducing the experimental $\lambda _{\max }^{a}$ values of KR2, BPR, ChR2-C128T, ChR2 and ChR_C1C2_ is provided in Section 3.2 of Ref. [69] and Section 3.3 of Ref. [43].

Recently, ${a}\text {-ARM}$ was applied to (1) reproduce the $\lambda _{\max }^{a}$ of WT KR2 and 19 mutants [35] (see Fig. 11a) and (2) to gain further insights into the origin of red- or blue-shifting. As observed in Fig. 11d, e, the performance of ${a}\text {-ARM}$ reported for the benchmark set (see above), is maintained when modeling a set of mutants that feature $\lambda _{\max }^{a}$ spanning a red-to-blue range going from 545 nm (54.5 kcal mol$^{-1}$, 2.36 eV) to 515 nm (57.0 kcal mol$^{-1}$, 2.47 eV). Furthermore, ${a}\text {-ARM}$ demonstrated to be useful to generate models that reproduce blue- or red-shifting effects observed experimentally (see Ref. [35]). The final $\text {S}_{0}$ optimized equilibrium structures can be then used for further excited-state optimizations (see Sect. 6.2). Indeed, some of the $\text{ARM QM/MM model}$s produced have been used as input for sophisticated constant-pH dynamics [75], the simulation of one-/two-photon absorption spectra [40], and in combined computational/experimental studies on color tuning possibilities in KR2 [35].

Accordingly, and as stated in Sect. 3.2, we claim that the ${a}\text {-ARM}$ protocol, in its current version, does not represent a predictive tool, but rather is designed to produce models for rhodopsins, which structure was obtained from either X-ray crystallography or comparative modeling, useful for reproducing and explaining the origin of trends in spectroscopic/photochemical properties (e.g., between sequence variability and function) appearing from sets of experimental data.

### Limitations and Pitfalls of *a*-ARM

Despite the encouraging outcome of the photochemical studies based on ${a}\text {-ARM}$ (as previously mentioned), additional work is necessary to generate a tool that can be systematically applied to larger arrays of rhodopsins. The following main issues, in part anticipated above, have to be tackled to improve the input file generator phase:

- Assignment of the protonation states: there are two main aspects that limit the confidence in the automation of the ionizable state assignment described in Refs. [43, 69]. The first is that, due to the fact that the information provided by PROPKA [76] is approximated, the computed p$K_a^{\text {Calc}}$ value may, in certain cases, be not sufficiently realistic. The second aspect regards the assignment of the correct tautomer of histidine. *a*-ARM uses as default the histidine dipeptide (HID) tautomer (deprotonated $\delta$-nitrogen) for the automatic assignment, or allows the user to choose between the three possible tautomers for a “not-automated selection. Therefore, when possible, the user should collect the available experimental data and/or inspect the chemical environment of the ionizable residues including the histidines, and propose the appropriate tautomer [69]. Alternatively, one has to systematically examine all sensible choices, which may not always be feasible.
- Automatic construction of comparative models: since rhodopsin structural data are rarely available, it would be important to investigate the possibility of building, automatically, the corresponding comparative models. With such an additional tool, one could achieve a protocol capable of producing QM/MM models starting directly from the constantly growing repositories of rhodopsin amino acid sequences. This target is currently pursued in our laboratory.
- Automatic prediction of side-chain conformation for mutants: recent efforts have been directed to achieve a successful technology for systematically predicting mutant structures, which provides a superior level of accuracy of the *a*-ARM models than that proposed in Ref. [69].
  More specifically, the mutations routine that used a backbone-dependent rotamer library (i.e., SCWRL4 [77]) was replaced by a software based on comparative modeling (i.e., MODELLER [78]) (see Fig. 8b). The description of the new approach as well as an example that illustrates its effectiveness for mutating a specific position with each of the 20 essential amino acids, are provided in Ref. [35] and summarized in Sect. 4.5.
- Insufficient description of possible cavity rearrangements after mutation: the updated procedure described in Ref. [35] for modeling the side-chain conformation (see point above) comprises a short MD, where the introduced side-chain is allowed to relax, whereas the rest of the cavity residues, water molecules, chromophore and protein environment remain fixed at the crystallographic/comparative structure (see Supplementary Note 13 in Ref. [35]).
  Notwithstanding the following, more sophisticated $\text{MD}$ step related to the cavity residues (see Sect. 3.4.1), a proper description of the impact of the new side-chain on the protein environment is lacking, due to a not sufficient description of possible local steric/electronic rearrangements of those residues of the chromophore cavity surrounding the mutated one.
- Mutations only allowed in the chromophore cavity: currently, ${a}\text {-ARM}$ only allows mutations of residues that belong to the chromophore cavity sub-system, as well as backbone relaxation is not allowed. The latter is to ensure that, during the QM/MM model generator phase, the geometry of the new modeled side-chain as well as the sidechain of its neighbors (belonging to the chromophore cavity) can be readjusted during the 1 ns GROMACS MD step, while assuming that the general structure of the protein is conserved.
- Lack of a predictive tool for mutants generation: the fact that the mutants generator relies on the use of experimental data to select the correct rotamer limits the usability of the protocol, which cannot be considered as a predictor tool.

### Recent Updates and Improvements

**Fig. 12:**
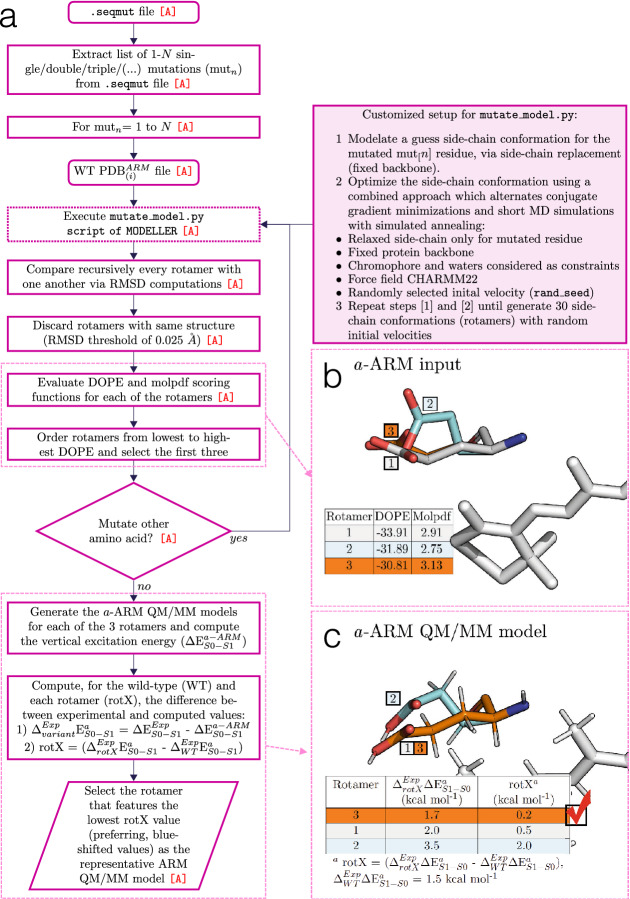
General workflow of the novel side-chain generator. **a** Modified routine for the mutants generator of ${a}\text {-ARM}$, based on Modeller. This procedure is used to model, e.g., the side-chain of the E219 residue of the KR2 rhodopsin, as shown in panels **b**, **c**. **b** First, the discrete optimized protein energy (DOPE) and molpdf scoring functions for all the possible rotamers are evaluated and the three best values are ranked. **c** Then, the a-ARM QM/MM model for each rotamer is generated and the rotamer model featuring the lowest difference in vertical excitation energy with respect to experimental data (rotamer 3) is selected Adapted with permission from [35], open access under a CC BY license (Creative Commons Attribution 4.0 International License)

In silico modeling of point mutations in proteins relies on the selection of a robust methodology for the prediction of the side-chain conformation of the replaced amino acid [77, 79–90]. Both *original* [59] and *advanced* [43, 64, 69, 91] versions of the ARM protocol use the software SCWRL4 [77] to predict the side-chain conformation of the mutated residues. This approach is based on backbone-dependent rotamer libraries (from public databases of experimentally resolved protein structures), and was found adequate for the production of single, double and triple point rhodopsin mutants. This was demonstrated by studies carried out by some of the authors on mutants of bovine rhodopsin (Rh) [59, 69], Anabaena Sensory rhodopsin (ASR) [42, 59], bacteriorhodopsin (bR) [74] and KR2 rhodopsins [92].

Recently, some of the authors reported the first attempt to use $a$-ARM model building to systematically and exhaustively mutate a single residue [35]. More specifically, they attempted, unsuccessfully, to perform single point mutations of KR2 at the P219 location near the $\beta$-ionone ring of the $r\text{PSB}$ via SCWRL4 modelling. Despite the encouraging results reported in Ref. [42] for the cases P219A, P219G and P219T, the authors found that, for larger side-chains SCWRL4 generated conformers sterically clashing with either the $r\text{PSB}$ or neighboring amino acids.

After examining the tools available for side-chain predictions (see, for instance, Ref. [79]) and evaluating them in terms of performance and accessibility as command-line tools, the authors of Ref. [35] modified the mutations routine (see Section 2.2.5. in Ref. [69]) by substituting SCWRL4 with MODELLER [78]. This alternative approach allows the production of mutants suitable for the prediction of absorption wavelengths in either an automatic or a computer-aided semi-automatic fashion.

Figure 12a illustrates the general workflow of the proposed subroutine, which replaces Step 3 of the input file generator phase of ${a}\text {-ARM}$ (see Fig. 8a). At input level, each point mutation is generated via a customized version of the $\texttt {mutate\_model.py}$ routine implemented in Modeller, where the conformation of the modeled side-chain is optimized using a conjugate gradient method, and refined using a short MD ($^{\text {mod}}$).

Briefly, as reported in Ref. [78], the mutate$\_$model.py script has been designed to model point mutations via side-chain replacement in a fixed environment, assuming that single mutations do not generally determine deep conformational changes of the protein backbone. Accordingly, and consistently with the structurally “conservative” approach of the ${a}\text {-ARM}$ protocol (see Sect. 3.3), our methodology replaces only the side-chains of the mutated residues keeping the backbone atoms at fixed positions. In order to sample the conformational space of a mutated residue more extensively. and evaluate its effect on the vertical excitation energy ($\Delta \text {E}_{S1-S0}^{a}$), the new customized setup produces 30 rotamers of the same mutated side-chain by providing the script with different initial seeds (i.e., initial velocities) for the MD$^{\text {mod}}$ run. The obtained rotamer structures are compared with each other, in terms of root mean square deviation, and discarded if found less than 0.025 Å from another. Although not particularly efficient, this procedure allows for the quick selection of a set of non-redundant rotamers for a single mutant, which are evaluated using the scoring function discrete optimized protein energy (DOPE) [93], implemented by MODELLER, and ranked from lowest to highest. The ARM input for the three highest DOPE scored mutated side-chain rotamers is completed by phase I of the ${a}\text {-ARM}$ protocol, and their ARM QM/MM models are produced using phase II (see Fig. 8). The corresponding computed $\Delta \text {E}_{S1-S0}^{a}$ is then used to evaluate the performance of different rotamers of the mutated side-chain in reproducing the experimental trend in line with the WT, leading to the selection to the conformer (rotamer) that better agrees with experimental data. Figure 12, panels b and c illustrate an example of the procedure for selecting a rotamer from three evaluated models. Further deatils can be found in Supplementary Note 13 in the supporting information of Ref. [35].

Although this approach relies on experimental information and does not represent a predictive tool, it automates the side-chain conformation selection during the construction of mutant QM/MM models.

## Web-ARM, a Web-Based Interface to ARM

(Most of the content of this section is reproduced/adapted with permission from [74]. Copyright 2020 American Chemical Society).

### Interface Features

**Fig. 13:**
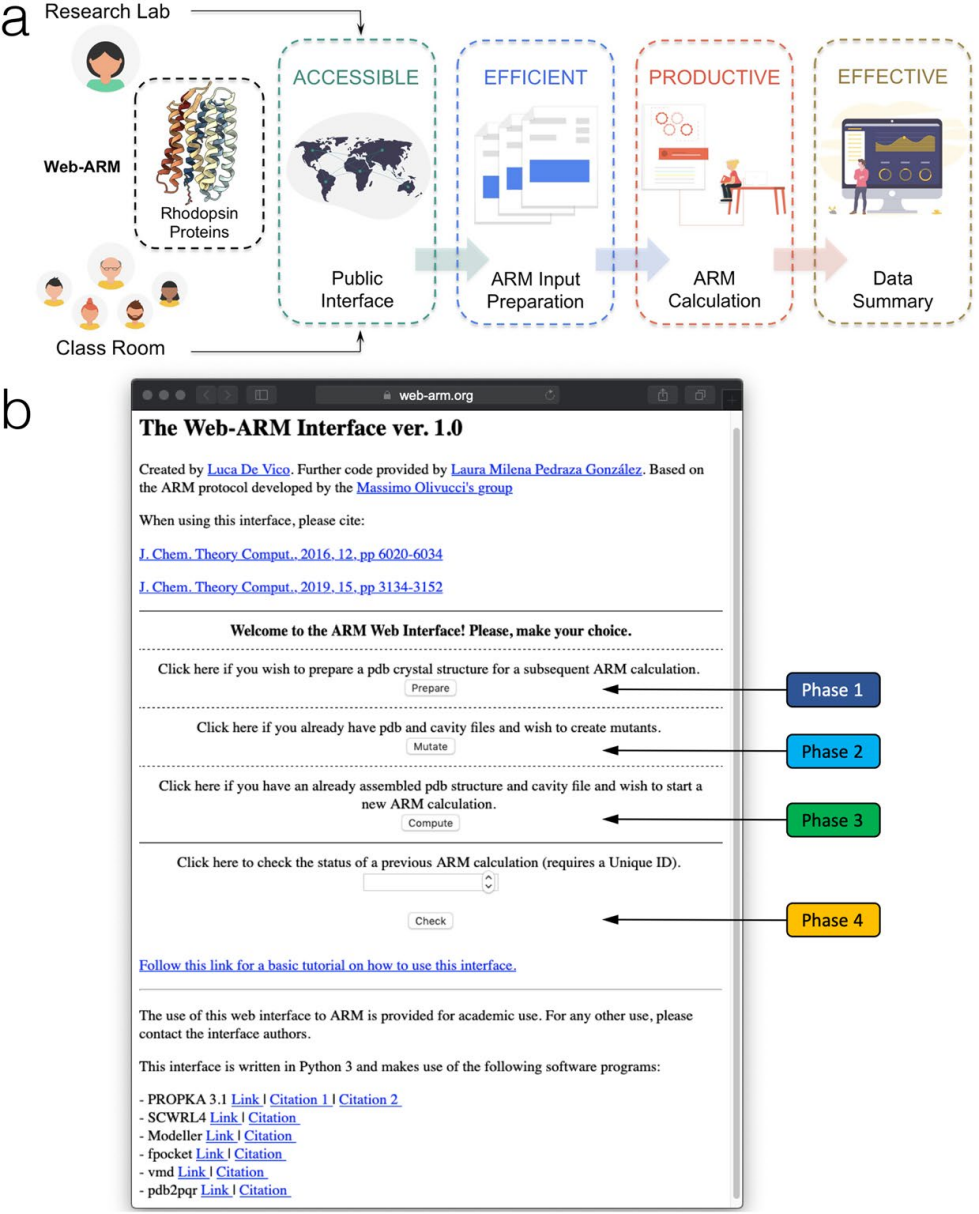
General overview of the Web-ARM interface. **a** Main features and **b** home page of the Web-ARM interface Adapted with permission from [74]. Copyright 2019 American Chemical Society

The ${a}\text {-ARM}$ protocol (described in Sect. 4) and its most updated version PyARM, a Python-based software package (that will be presented in Sect. 6) represent an easy-to-use command-line interface directed to users (i.e., researchers, undergraduate students) familiar with the Linux environment. The latter is not due to a fact of usability, but rather to the technically complex initial setup of the package, since it requires the prior installation of several software and python dependencies. Moreover, it is recommended to install the PyARM package in a high-performance computer cluster, which is usually required to run the underlying computationally intensive tasks (i.e., $\text{MM}$, $\text{MD}$, $\text{QM/MM}$), rather than on a local personal machine.

The use of a user-friendly interface accessible through the web and together with the provider computational resources, avoids dealing with complicate installations and the need for local computer facilities. This would mean accessing a simple, computationally fast and automated construction and analysis of rhodopsin $\text{QM/MM}$ models, fit for an interdisciplinary community that is interested mostly in actual applications, rather than methodological development.

Accordingly, Ref. [74] reports the web version of the ${a}\text {-ARM}$ protocol, that is the $\text{Web-ARM}$ interface. $\text{Web-ARM}$ is a user-friendly interface written using Python 3, available at the following address: web-arm.org. Therefore, the potential user only needs to use an up-to-date browser on any operating system (i.e., Linux, macOS, Windows, Android, iOS) and platform to take advantage of the ${a}\text {-ARM}$ protocol.

In order to generate an $\text{ARM QM/MM model}$, $\text{Web-ARM}$ goes through the four phases explained in Section 2.1 of Ref. [74], and illustrated in Fig. 13. As described for the command-line version, the procedure starts with the initial structure of the rhodopsin variant and finishes with the generation of the $\text {S}_{0}$ equilibrium geometry along with the calculations on absorption properties. During such a procedure, the interface gives the user enough flexibility to generate either ${a}\text {-ARM}$ $_\text {default}$ or ${a}\text {-ARM}$ $_\text {customized}$ inputs (see Sect. 4.1), the former automatically and the latter by modifying some of the default choices. This is made on top of the implementation of the input file generator inside the framework of the web interface. Then, the so-generated $\text{ARM input}$ is used to compute a $\text{QM/MM}$ model, by using the QM/MM model generator. The $\text{Web-ARM}$ internal driver takes care of performing all the necessary steps, as well as submitting the calculations to the dedicated computational facilities.

One feature of the interface is that, once a $\text{QM/MM}$ model is generated, the user is provided with a summary of all the relevant data (i.e., energetics, oscillator strengths), along with a downloadable file (in compressed format) containing the major output files. Further information, and a complete walk-through, are provided in a Tutorial that can be accessed/downloaded from the $\text{Web-ARM}$ main web page.

$\text{Web-ARM}$ is intended as both a research, as well as a teaching tool. Ref. [74] shows that the interface can systematically screen rhodopsin variants, and thus obtain a qualitative check prior to, e.g., an experimental study. The interface can also be used successfully in teaching and learning activities, e.g., to introduce students to the idea of $\text{QM/MM}$ models and corresponding computed data. Therefore, $\text{Web-ARM}$ is envisioned as a tool used in teaching and training, as well as by non-experienced users, as previously noted, mostly for bulk production. However, also an experienced computational chemist can take advantage of the web interface, to produce rhodopsin $\text{QM/MM}$ models in a standardized manner, being aware of the documented accuracy and rate of success. Of course, one, possibly very useful, application of such a model is to provide high quality guesses for more sophisticated subsequent calculations, e.g., as a starting substrate to which apply further, high-level refinement methods.

In conclusion, by using $\text{Web-ARM}$ both junior researchers and trainees will be able to perform meaningful $\text{QM/MM}$ calculations focusing on the underling research targets, methodological concepts, and data analysis, while remaining confident that the calculations are internally consistent.

### Limitations and Future Development of Web-ARM

**Fig. 14:**
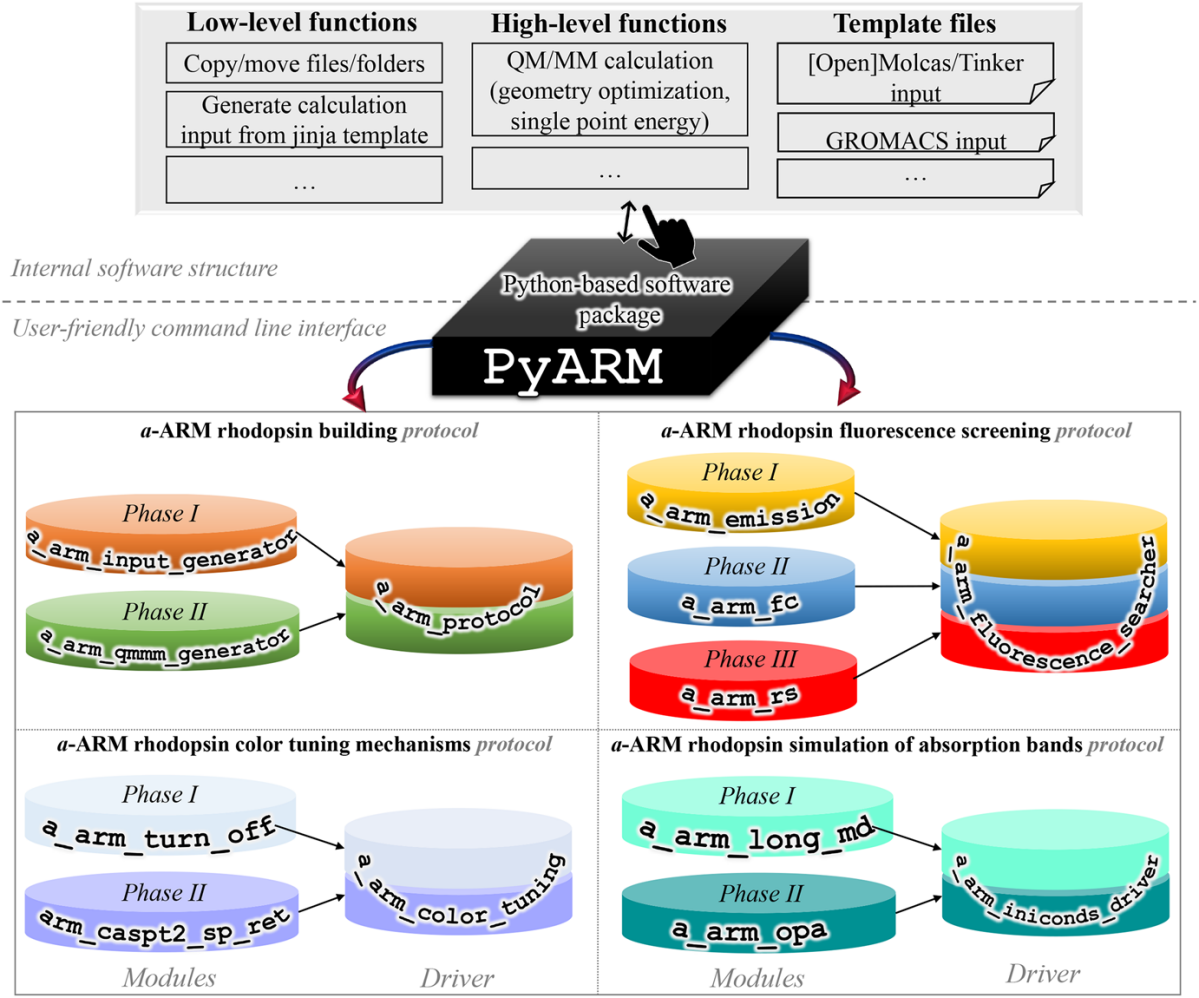
Representation of the contents of the PyARM software package. The package contains various modules, which can be steered using a driver. Modules make use of high- and low-level functions, as well as template files that are also an integral part of the package

- Limited computational resources: before using the $\text{Web-ARM}$ interface, the user is asked to provide an email address to be registered into our database. Registered users are allowed to build as many concurrent $\text{ARM QM/MM model}$s as wished (default 10). However, given to the present threshold in the host available computational resources, guest users are allowed to build only one $\text{ARM QM/MM model}$ model at a time on the developer’s dedicated resources.
- Technical issues: the Tutorial of the $\text{Web-ARM}$ interface reports on possible errors or issues in the execution of the interface, and how to solve or avoid them.
- Current and future implementation: presently, the capability of the $\text{Web-ARM}$ interface is limited to the construction of ground-state models. Future work will implement inside the interface all of the features of the PyARM software package, illustrated in Fig. 14.

## PyARM

### Package Description

PyARM is a user-friendly, open-source Python-based software tool designed to facilitate the systematic, reproducible and congruous $\text{QM/MM}$ modeling/analysis of photoexcited states of rhodopsin proteins. More specifically, PyARM is a development platform that implements high-level algorithms associated to specialized $\text{QM/MM}$ protocols, under the framework of the ${a}\text {-ARM}$ protocol [43, 59, 64, 69, 74, 91].

The package structure represents a collection of hierarchical instances here defined as: scripts, basic low-level functions, general high-level functions, modules, drivers and templates. Table 1 provides a technical definition of each of these terms.

**Table 1:** Different instances of the PyARM framework

Instance	Definition
Script	Python file that is intended to be executed directly. This means that scripts will often contain code written outside the scope of any classes or functions
Basic low-level function	Basic, importable function that performs a single, simple and generic task. Example: Copy/move files, create folders, calculate energy gaps, generate input file
General high-level function	General function that makes use of basic low-level functions to perform a more complex task. Can be used either as importable or as stand-alone by parsing arguments in the terminal. Example: quantum chemistry calculation
Module	Script that makes use of both basic low-level and general high-level functions to perform a complex task (i.e., semi-protocol) and that requires different stages. Can be used either as importable for the drivers or as stand-alone by parsing arguments in the terminal. Example: Input file generator ${\texttt {<<a\_arm\_input\_generator>>}}$
Driver	Script that makes use of the modules to implement a complex protocol. Works as stand-alone by parsing arguments in the terminal. Example: $a$-ARM model building ${\texttt {<<a\_arm\_protocol>>}}$
Package	Collection of related modules that work together to provide certain functionality. These modules are contained within a folder (${\texttt {/src/}}$) and can be imported just like any other modules. This folder contains a special $\texttt {\_\_init\_\_.py}$ file that tells Python it’s a package, potentially containing more modules nested within subfolders. Example: $\texttt {<<\texttt {PyARM}>>}$

Figure 14 depicts the general overview of PyARM, showing how all components of the package are modular, i.e., independent of the context in which they are used. The characteristics of the drivers and package create an application-programming tool, capable of making complex workflows/protocols available to users with minimal programming knowledge. Indeed, the inclusion of new protocols (i.e., modules and drivers) can be achieved easily with only a minimal modification of the code. In this regard, the package includes a programming interface for parsing user input, and retrieving and storing specific data. Therefore, a user can simply use a module to perform a given type of application (e.g., perform a geometry optimization) or use a driver to launch a protocol connecting different modules and, thus, executing more complex applications (e.g., generate a $\text {S}_{0}$ or $\text {S}_{1}$ $\text{QM/MM}$ model, locate a fluorescent rhodopsin, compute an absorption band, etc.). Nevertheless, an experienced user can also create a new driver, capable of performing a new type of application/analysis based on ${a}\text {-ARM}$ models and, therefore, within the limits of their accuracy [59, 69, 74].

Furthermore, the modular framework of the package, which was envisioned initially for the study of natural rhodopsins, presents a flexible architecture suitable to be generalized to other photoreceptors including synthetic light-responsive proteins. As a first step in such a direction, the current implementation is suitable for the study of rhodopsins featuring artificial retinal-like chromophores, which is currently pursued in our group.

### Current ARM-Based QM/MM Protocols

Presently, PyARM contains four different $\text {ARM}$-based fully automatic protocols, each implemented as a general driver (i.e., user-friendly one-click command-line interface), for the $\text{QM/MM}$ modeling of rhodopsin electronically excited states. A brief description of each of these protocols, illustrated in Fig. 14, is provided below.

1. Protocol: $a$-ARM model building protocol: ARM with chromophore cavity generation, ionization state selection, and external counterion placement.
  Driver: $$\texttt {a\_arm\_qmmm\_protocol}$$
    Phase I: Input file generator. Module: $\texttt {a\_arm\_input\_generator}$ (see Fig. 8a)
    Phase II: $\text{QM/MM}$ model generator. Module: $\texttt {a\_arm\_qmmm\_generator}$ (see Fig. 8b)
  Description: The first step towards the standardization and full automation of the $original \,\text{ARM}$ was the development of ${a}\text {-ARM}$. This updated version not only overcame the automation drawbacks of the original version, but also included significant methodological/computational improvements. The achieved level of automation is accompanied by other features, such as speed in preparing the model building input, and standardization and reproducibility of the final model when operated by different users. In fact, the time required for preparing the input for the QM/MM model construction is reduced from around 3 h to less than 5 min (user time), with respect to the original ARM protocol. This is a consequence of the automation of the different preparatory steps, thus avoiding the user manipulation of text files and/or visualization of chemical structures. More specifically, a Python-based automatic input file generator subroutine was written to automate the assignment of the residues defining the chromophore cavity, including the chromophore linker and counter-ions, the protonation state of ionizable residues and, finally, the unambiguous placement of cytoplasmic and extracellular counter-ions. The benchmark calculations demonstrated that the resulting models for several rhodopsin sets are accurate enough for reproducing experimental trends in vertical excitation energy (i.e., the computed vertical excitation energies have an error bar of less than 4.0 kcal mol$^{-1}$ blue-shifted), as well as transferability to rhodopsins of very different sequence. This protocol has been further described in Sect. 4.
2. Protocol: Automated Analysis of Color Tuning Mechanisms in Rhodopsins.
  Driver: $$\texttt {a\_arm\_color\_tuning}$$
    Phase I: Electrostatic effects. Module: $\texttt {a\_arm\_turn\_off}$ (see Sects. 6.4.3 and 6.4.4)
    Phase II: Steric effects. Module: $\texttt {arm\_caspt2\_sp\_ret}$ (see Sect. 6.4.2)
  Description: This protocol provides a tool to study how the protein environment (i.e., protein sequence) modulates the absorption wavelength of the retinal chromophore and, in turn, the color of the protein. In other words, it performs rhodopsin color tuning analysis so as to reveal steric and electrostatic effects between the retinal chromophore and the surrounding cavity amino acid residues. This tools aids the mechanistic description of the ways amino acid residues influence the photophysical properties of the retinal chromophore. This, in turn, builds up a reference book for helping the tuning of rhodopsin cavities (i.e., via point mutations) towards a desired effect (e.g., blue or red shifted wavelength). This protocol will be further described in Sect. 6.4.
3. Protocol: Automated Simulation of Absorption Bands and light-induced Dynamics of Rhodopsins.
  Driver: $$\texttt {a\_arm\_iniconds\_driver}$$
    Phase I: Long MD from a-ARM QM/MM model. Module: $\texttt {a\_arm\_long\_md}$
    Phase II: Simulation of one photon absorption spectrum and light-induced dynamics. Module: $\texttt {a\_arm\_opa}$
  Description: This protocol allows the automatic simulation of one photon absorption (OPA) spectra, and the subsequent calculation of the initial conditions necessary for the study of the excited state dynamics, also allowing estimation of the photoisomerization quantum yield (QY). In other words, this tools provides another link between experimental quantities and simulated results. The photoisomerization QY, in particular, is of importance when interested in devising rhodopsins that are either particularly reactive or, contrarily, extremely fluorescent. This protocol will be the subject of a future publication.
4. Protocol: Automated QM/MM Model Screening of Rhodopsin Variants Displaying Enhanced Fluorescence.
  Driver: $$\texttt {a\_arm\_fluorescence\_searcher}$$
    Phase I: Location of the S$_1$ $\text{fluorescent excited-state (FS)}$. Module: $\texttt {a\_arm\_emission}$
    Phase II: Computation of Franck–Condon (FC) trajectories. Module: $\texttt {a\_arm\_fc}$
    Phase III: Calculation of the S$_1$ photoisomerization path (RS). Module: $\texttt {a\_arm\_rs}$
  Description: This protocol is designed for gaining insights into the possible molecular-level mechanisms behind fluorescence enhancement, when the fluorescent species correspond to the initial dark state of the rhodopsin photocycle. It is composed of three different phases able to categorize a rhodopsin as dim- or enhanced-fluorescent, with respect to a reference. This allows the fast and systematic in silico screening of potentially hundreds of rhodopsin mutants. Furthermore, each phase provides information (i.e., properties such as emission wavelength, excited state lifetime (ESL), $\text{energy isomerization barrier}$ $(\text {E}^{f}_{S1})$, fluorescence quantum yield ($\phi ^{f}$) and structural parameters) that allows to elucidate and understand the factors determining rhodopsin fluorescence and, possibly, learn how to modulate it, with the ultimate goal of designing fluorescent candidates in silico for applications in, e.g., optogenetics [94]. This protocol will be the subject of a future publication.

### **PyARM** Technical Details

#### **PyARM** Default Parameters

The setup for all the MD (see Sect. 3.4.1) and $\text{QM/MM}$ (see Sect. 3.4.2) calculations performed by the different modules and drivers of PyARM (Sect. 6.2), is consistent with that reported for the ${a}\text {-ARM}$ protocol in Refs. [43, 59, 64, 69] (see also Supplementary Notes 2 and 3 of Ref. [35]).

As described in  Sect. 3.4.2, the default parameters for QM/MM calculations correspond to 2-roots single-state CASSCF(12,12)/AMBER optimization and state-average 3-roots CASPT2(12,12)/6-31G(d) energy correction. Such values for the active space and number of roots were selected based on benchmark calculations [59]. While the latter is not a modifiable parameter, the former could be modified via command-line arguments; however, it is strongly recommended to keep the default values.

As shown in Sect. 6.2, each instance of PyARM (see Table 1) is executed via a command-line interface, which contains a help menu facility that provides descriptions and syntax for the default parameters and how to modify them. Some of these parameters are listed below. Note that it is possible (but not recommended) to customize all the parameters for the MD run.

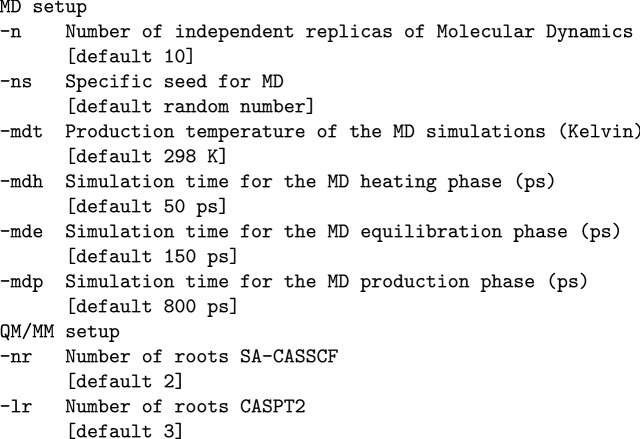



#### **PyARM** Installation

The PyARM code can be obtained by contacting the authors. PyARM relies on the following programs being already present and properly installed: Molcas ver. 8.4 or OpenMolcas [62, 64]; TINKER ver. 6.3 (with specific $\text{QM/MM}$ patches) [63]; PROPKA ver. 3.1 [76]; PutIon [59]; GROMACS ver. 4.5.5 [61]; DOWSER [60]; PDB2PQR [95, 96]; Fpocket [97]; Modeller ver. 9.18 [78]. Furthermore, PyARM necessitates of a Python 3.x installation including the following modules: OpenBabel [98]; MDAnalysis [99, 100]; Matplotlib [101]; pandas [102]; NumPy [103]; SciPy [104]; Jinja2; Python-crontab; PyYAML; TextTable; cclib [105], GromacsWrapper.

PyARM contains a configuration file, the contents of which point to the installation path of the aforementioned programs. Submission template files are also provided for each type of calculation. These templates have to be tailored to the specifics of the computer cluster queuing system. Once these files are in place, and the user has write permissions for the Python installation, PyARM is installed as a normal Python 3.x package.

#### **PyARM** Tailoring

The modular nature of PyARM allows the relatively easy implementation of changes in the various drivers, as well as introducing a new driver. As a hypothetical example, ${a}\text {-ARM}$ models have been used to compare excitation energy data obtained using CASPT2, MS-CASPT2 and MC-PDFT levels of theory [106]. The introduction of a different level of theory to evaluate excitation energies would imply using a new high-level function (Table 1) that performs such calculation, which in turn is based on a new template input file.

The new high-level function would be used by one or more modules, which in turn would belong to any number of drivers. If the user would like to simply swap level of theory, they would need to change the call to the original high-level function in each module with the newly made one. Otherwise, the user can simply create copies of the desired modules and drivers, which makes use of the new high-level function.

Similarly, a new driver could be implemented, taking advantage of the already present modules and functions as much as possible. For example, one could introduce a new, hypothetical, driver to locate the possible photoproduct structure of a given rhodopsin. Such a driver would combine the machinery of the previously mentioned $\texttt {a\_arm\_fluorescence\_searcher}$, but extending the $\texttt {a\_arm\_rs}$ module (i.e., making a new module) to perform a ca. 180 degrees isomerization, followed by a S$_0$ geometry optimization and energetic evaluation. In this case, all necessary high-level functions and templates are already present, they just have to be combined in a different way.

### Color Tuning Analysis in Terms of Steric and Electrostatic Effects

(Part of the content of the next three sections is reproduced/adapted with permission from [35], open access under a CC BY license (Creative Commons Attribution 4.0 International License)).

**Fig. 15:**
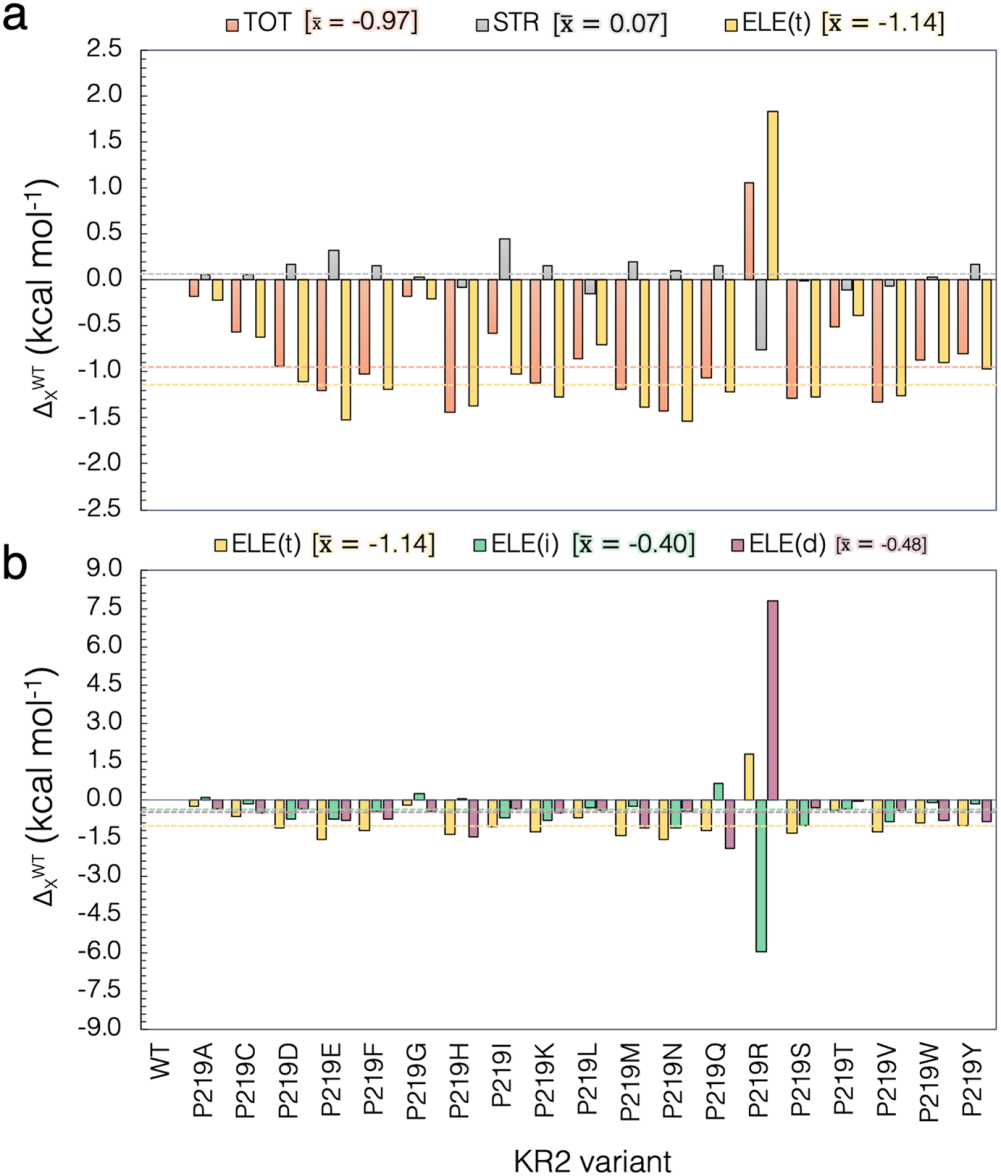
Steric and electrostatic contributions to the vertical excitation energy. **a** Total ($\Delta \Delta$E$_\text {S1-S0}^\text {TOT}$), steric ($\Delta \Delta$E$_\text {S1-S0}^\text {STR}$) and electrostatic ($\Delta \Delta$E$_\text {S1-S0}^\text {ELE(t)}$) contributions to the interaction of the retinal with the protein environment for the possible P219X mutations. **b** Decomposition of the total electrostatic effects on its indirect ($\Delta \Delta$E$_\text {S1-S0}^\text {ELE(i)}$) and direct ($\Delta \Delta$E$_\text {S1-S0}^\text {ELE(d)}$) components. The dashed lines and corresponding numerical values refer to the weighted average values (${\overline{x}}$) of the 18 residues of the red-shifted cluster exclusively (that is, excluding P219R), presented in square parenthesis. Reproduced with permission from [35], open access under a CC BY license (Creative Commons Attribution 4.0 International License)

As an example of a possible usage of a driver and a module as found inside PyARM, this section presents the driver ($\texttt {a\_arm\_color\_tuning}$) behind a common kind of analysis [35, 42], which aims at discerning the contribution of nearby cavity residues onto the computed excitation energy $\Delta \text {E}_{S1-S0}^{a}$ of the $r\text{PSB}$, and assign them as blue- or red-shifting. The shifting contributions are assessed in terms of both electrostatic effects onto the charged $r\text{PSB}$ (Sect. 6.4.3), and steric effects distorting the geometry of the retinal chromophore (Sect. 6.4.2). This assessment is performed based on quantities defined in Sect. 6.4.1. Figure 15 presents the results of using this analysis, as found while performing all possible mutations (P219X) for the KR2 rhodopsin [35]. Finally, Sect. 6.4.4 presents a simpler possible use of the module $\texttt {a\_arm\_turn\_off}$ to analyze electrostatic contributions from cavity residues onto the $r\text{PSB}$.

#### Computed Quantities

In Ref. [35], the authors define three fundamental quantities ($\Delta \Delta$E$_\text {S1-S0}^\text {TOT}$, $\Delta \Delta$E$_\text {S1-S0}^\text {STR}$, and $\Delta \Delta$E$_\text {S1-S0}^\text {OFF}$) whose values are a function of either the structural (both at the chromophore and protein cavity levels) or electrostatic changes of each mutant with respect to WT, using as an illustrative example the mutants P219X of the $\text{KR2}$ rhodopsin (Fig. 11c).

- $\Delta \Delta$E$_\text {S1-S0}^\text {TOT}$ is the “Total” excitation energy change. It is computed directly as the difference between the QM/MM computed vertical excitation energies of mutant and WT models.
- $\Delta \Delta$E$_\text {S1-S0}^\text {STR}$ is the “Steric component” of $\Delta \Delta$E$_\text {S1-S0}^\text {TOT}$. It is computed directly as the difference between the QM/MM vertical excitation energies of the isolated retinal chromophores, however retaining the geometries as if inside the protein environment, between mutant and WT.
- $\Delta \Delta$E$_\text {S1-S0}^\text {OFF}$ is used to quantify the “indirect” electrostatic component (see below) of $\Delta \Delta$E$_\text {S1-S0}^\text {TOT}$. It is computed as the difference between the vertical excitation energy of the mutant and WT obtained after having switched off (turned to zero) the charges of residue 219. $\Delta \Delta$E$_\text {S1-S0}^\text {OFF}$ can also be used directly to analyze, qualitatively, the red- or blue-shifting role of each of the residues of the chromophore cavity for a determined rhodopsin (Turn-Off Module). In such a case, it is computed as the differences between the vertical excitation energy of the rhodopsin before and after having switched off (turned to zero) the charges of specific residues.

As we will see in the following, using these quantities, we can compute three additional components.

- $\Delta \Delta$E$_\text {S1-S0}^\text {ELE(t)}$ is the “Total electrostatic component” that is indirectly computed as the difference between the Total and the Steric components above ($\Delta \Delta$E$_\text {S1-S0}^\text {ELE(t)}$ = $\Delta \Delta$E$_\text {S1-S0}^\text {TOT}-\Delta \Delta$E$_\text {S1-S0}^\text {STR}$) for each mutant. As specified in Sect. 6.4.2, $\Delta \Delta$E$_\text {S1-S0}^\text {ELE(t)}$ can be decomposed into two parts:
  - $\Delta \Delta$E$_\text {S1-S0}^\text {ELE(i)}$ is the “Indirect electrostatic component” that is indirectly computed in two steps by first computing the differences between the vertical excitation energy of the mutant and WT obtained after having switched off (turned to zero) the charges of residue 219, and then by subtracting from such difference the steric effect $\Delta \Delta$E$_\text {S1-S0}^\text {STR}$ defined above.
  - Finally, $\Delta \Delta$E$_\text {S1-S0}^\text {ELE(d)}$ is the “Direct electrostatic component” that is computed indirectly as $\Delta \Delta$E$_\text {S1-S0}^\text {ELE(d)}$ = $\Delta \Delta$E$_\text {S1-S0}^\text {ELE(t)}$ - $\Delta \Delta$E$_\text {S1-S0}^\text {ELE(i)}$.

#### Steric Effects

In the context of the protocol, by “steric effects” we mean “indirect” or “geometrical” effects, i.e., the change in excitation energy of a chromophore due to a change in the minimum geometry. In turn, this change in geometry of the $r\text{PSB}$ could be induced by both steric and electrostatic factors. Such effects are investigated by analyzing how the retinal chromophore is structurally modified by mutations near the $\beta$-ionone or near the Schiff base linkage (see Fig. 5).

As discussed in Ref. [35], such structural rearrangements of the $r\text{PSB}$ may be due to different factors, such as a simple effect induced by the side-chain replacement, a different charge distribution due to changes in protonation states for ionizable residues, as well as water molecules addition/removal. Notice that steric effects were evaluated through an “atomistic” approach, focused on the changes in the $r\text{PSB}$ geometrical and electronic structure, and therefore not directly related to steric effects evaluated on the basis of the changes in residue volume [35, 92].

#### Electrostatic Effects

As mentioned above, the total electrostatic effect ($\Delta \Delta$E$_\text {S1-S0}^\text {ELE(t)}$) can be decomposed in two parts: (1) the first can be considered as a direct component ($\Delta \Delta$E$_\text {S1-S0}^\text {ELE(d)}$) due to the variation in number, magnitude, and position of the point charges of mutated residue caused by the P to X replacement, and (2) a more indirect component ($\Delta \Delta$E$_\text {S1-S0}^\text {ELE(i)}$) produced from the reorganization of the local environment and hydrogen bond network induced by the same replacement and due to the fact that conserved residues and water molecules change in position or orientation. Moreover, as discussed in Ref. [35], possible changes in protonation states of conserved residues, induced by P to X replacement, have a major contribution to the indirect component. Figure 15 shows the contributions due to different effects (steric and electrostatic) and components.

#### Turn-Off Module

As a final example, we present a possible usage of the $\texttt {a\_arm\_turn\_off}$ module, which is part of the $\texttt {a\_arm\_color\_tuning}$ driver (Phase I). As previously stated (Table 1), modules can be used also independently from the parent driver. When used as a stand-alone script, the command-line menu of the module provides four different options to turn-off residues in the chromophore cavity, with different scopes, as follows:

1. $\texttt {<cav>}$: Turn off the charges of EACH of the residues in the cavity, independently. For instance, if the cavity is formed by *N* residues, the output will be *N* single calculations.
2. $\texttt {<cav\_all>}$: Turn off the charges for ALL the residues in the cavity, simultaneously. Therefore, the output will be a single calculation.
3. $\texttt {<single>}$: Turn off the charges for ONE specific residue given by the user.
4. $\texttt {<multiple>}$: Turn off the charges for a list consisting of MULTIPLE residues, given by the user, simultaneously. Therefore, the output will be a single calculation.

As an example of a possible analysis, we envision the use of the module with the first option ($\texttt {<cav>}$), to map out the expected electrostatic influence of cavity residues onto the $\Delta \text {E}_{S1-S0}^{a}$ of the $r\text{PSB}$. Such analysis could drive subsequent mutation tests, such as all possible mutations performed onto the residue responsible for, e.g., the largest or smallest shift.

### **PyARM** Current Accuracy and Drawbacks

A number of limitations of ${a}\text {-ARM}$ have already been described in Sects. 3.5, 4.4 and 5.2. Due to their simplified definition, $\text{ARM}$ models are more exposed to potential pitfalls than more complex QM/MM models. Such possible pitfalls, that concern the QM/MM model generator phase, can be summarized as:

1. lack of a proper description of the protein environment (membrane + explicit solvent),
2. rigid protein backbone and non-cavity side-chains,
3. approximated protonation states for ionizable residues, and
4. missing description of any mutual polarization effects between the QM and MM sub-systems, that can be accounted for by polarizable embedding using a polarizable force field. Since polarizable force fields are technologies still under development in the QM/MM area (see, for instance Ref. [107]), we have not adopted/benchmarked them in this version of our specialized QM/MM models.

When considering points 1–4, the different properties computed by ARM are expected to be affected by a systematic error. Our current research is aimed at dealing with those points, while maintaining reasonable computational costs, or estimating the errors due to them. Nevertheless, according to the philosophy of the ARM protocol (Sect. 3.2), the main focus of ARM is the ability to reproduce property (especially $\lambda _{\max }^{a}$) and explain trends, rather than predicting their absolute values. The default model generation and subsequent customization revised above clearly shows that the models can fit the experimental results. In other words, the customization protocol searches for possible sources of systematic errors (i.e., different protonation state, different rotamer), due to points 1–4.

A trend deviation factor was computed [69], to evaluate the accuracy of the ${a}\text {-ARM}$ data in a given set of rhodopsins with available experimental data. Thus, a mean absolute error of 2.5 kcal mol^−1^ was found (Table S4 in the supporting information of Ref. [69]), when considering ${a}\text {-ARM}$_default_ results for the benchmark set of Fig. 11b. A smaller value of 0.4 kcal mol^−1^ (Supplementary Table 5 of Ref. [35]) was found for the coherent set of KR2 mutants presented in Fig. 11d. These numbers show the expected accuracy of PyARM in evaluating trends of photophysical properties of rhodopsin sets. Logically, a coherent set of mutants of the same rhodopsin shows a smaller deviation, with respect to a set of rhodopsins spanning different organisms.

Although outside the ${a}\text {-ARM}$ scope, it is possible to evaluate the accuracy of PyARM with respect to computing absolute absorbed wavelengths.^4^ For example, with respect to the data behind Fig. 11b (Table S3 in the supporting information of Ref. [69]), we found an average accuracy for the ${a}\text {-ARM}$_default_ protocol of 90%.

## Outlook and Concluding Remarks

In this review, we presented the ARM protocol, along with its past and current development and achievements. After a brief introduction to rhodopsins, their importance and current and potential technological uses, we presented the development of $\text{ARM}$. We saw how the protocol was thought from inception to be accurate enough to reproduce trends in photochemical properties (mainly $\Delta \text {E}_{S1-S0}^{a}$), that is points (1) and (2) of Sect. 3.5.

Most of the remaining points (3)–(5) were addressed through the subsequent development of the $\text{Updated version of the Automatic Rhodopsin Modeling protocol}\,{{a}\text {-ARM}}$, presented in Sect. 4. The major achievement of ${a}\text {-ARM}$ has been the complete automation of the input file generator and QM/MM model generator, and the corresponding coding as python, user-friendly, command-line interfaces. Such tools were also included in Sect. 5. The method was benchmarked (Fig.  11), and found reliable in obtaining photochemical properties of different rhodopsins, obtained from various life domains. Furthermore, the usage of the input file generator ensured reproducibility of the results, even when considering the two approaches ${a}\text {-ARM}$_default_ and ${a}\text {-ARM}$_customized_.

Finally, Sect. 6 presented the latest development of the ARM protocol, namely PyARM. As shown in Fig. 14, PyARM consists of different drivers capable of performing various actions and automatic analyses, combining several python modules, which in turn are composed of functions and templates (Table 1). The automation level achieved by PyARM allows the efficient generation of, e.g., all 19 possible mutants of a given residue (Fig. 11c). The concurrent preparation of many $\text{QM/MM}$ models allows, in principle, the study of rhodopsin mutants arrays. The completely modular architecture of PyARM will permit scaling up the code (i.e., introducing novel kinds of analyses), through the implementation of new drivers, alongside those described in Sect. 6.2.

Despite the encouraging outcome of the applications of the protocol, additional work is required, for providing the scientific community with a robust tool that can be applied systematically to the study and design of sizable rhodopsin arrays. Much of its future success will depend on further improvements in the construction of the ${a}\text {-ARM}$ model and, in particular, of rhodopsin mutants models. Some of the envisioned improvements are as follows:

- Since rhodopsin structural data are rarely available and still difficult to obtain experimentally, it would be important to integrate, in an automatic fashion, comparative modeling technologies in the protocol for ${a}\text {-ARM}$ model building. It is possible that with such tool one could achieve a protocol capable of producing more accurate $\text{QM/MM}$ models starting directly from the constantly growing repositories of rhodopsin sequences. This target is currently pursued in our laboratory.
- The above item is also related to the improvement of mutant models generation and screening. As a perspective of this work, we suggest to introduce a mutant generator routine using proper comparative (homology) modeling, instead of just modifying the mutated side-chain conformation locally, as seen in Sect. 4.5.
- Both previous items could also benefit from the introduction of a machine-learning or artificial intelligence (AI)-assisted protocol for more accurate structures or overall structure predictions.
- Methods for improving the prediction of the residue protonation states during the construction of $\text{ARM QM/MM model}$s appear of capital importance for increasing the accuracy (e.g., the percentage of success in reproducing trends in spectroscopic properties) of the final models. These are being presently investigated in our lab (see for instance a preliminary study in Ref. [75]).
- Currently, the $a$ ARM model building protocol is the only $\text{ARM}$-based tool implemented in the $\text{Web-ARM}$ interface and, therefore, accessible through the web. Future efforts will be devoted to the implementation of the four protocols described in Sect. 6.2 as utilities of the $\text{Web-ARM}$ interface.

Finally, long-term development goals include introducing new drivers for PyARM (possibly re-using some of the already existing modules and scripts) for novel kinds of analyses. It is our intention to pursue also the possibility of simulating the use of different retinal chromophores, either natural or synthetic. We foresee the possibility of having a generalized method capable of handling any kind of chromophore. Last but not least, we would like to extend the applicability of the protocol beyond rhodopsins, and be able to apply it to many (any) photoactive protein complex.

## Data Availability

The code can be obtained by contacting the authors.

## Footnotes

1 Part of a molecular entity consisting of an atom or moiety in which the electronic transition responsible for a given spectral band above 200 nm is approximately localized [4].

2 Classically, the Franck–Condon principle is the approximation that an electronic transition is most likely to occur without changes in the positions of the nuclei in the molecular entity and its environment. The resulting state is called a Franck–Condon state, and the transition, a vertical transition [4].

3 Reversible transformation of a molecular entity between two forms, A and B, having different absorption spectra, induced in one or both directions by absorption of electromagnetic radiation. The spectral change produced is typically, but not necessarily, of visible color and is accompanied by differences in other physical properties [4].

4 We here define accuracy as the absolute difference of computed and experimental absorbed wavelength, divided by the experimental number, in percentage. This is done for each of the *N*=10 replicas of a rhodopsin (Sect. 3.4.1) and then averaged. The final number is the average of the accuracy of all the considered rhodopsins.
